# Associations between synthetic phenols, phthalates, and placental growth/function: a longitudinal cohort with exposure assessment in early pregnancy

**DOI:** 10.1093/hropen/hoae018

**Published:** 2024-04-01

**Authors:** Nicolas Jovanovic, Vicente Mustieles, Marc Althuser, Sarah Lyon-Caen, Nadia Alfaidy, Cathrine Thomsen, Amrit Kaur Sakhi, Azemira Sabaredzovic, Sam Bayat, Anne Couturier-Tarrade, Rémy Slama, Claire Philippat

**Affiliations:** University Grenoble Alpes, Inserm U1209, CNRS UMR 5309, Team of Environmental Epidemiology Applied to Reproduction and Respiratory Health, Institute for Advanced Biosciences, Grenoble, France; University Grenoble Alpes, Inserm U1209, CNRS UMR 5309, Team of Environmental Epidemiology Applied to Reproduction and Respiratory Health, Institute for Advanced Biosciences, Grenoble, France; Instituto de Investigación Biosanitaria (ibs. GRANADA), Granada, Spain; CIBER de Epidemiología y Salud Pública (CIBERESP), Spain; Department of Radiology and Physical Medicine, University of Granada, Biomedical Research Center (CIBM), Granada, Spain; Department of Obstetrics/Gynecology and Fetal Medicine, Grenoble University Hospital, Grenoble, France; University Grenoble Alpes, Inserm U1209, CNRS UMR 5309, Team of Environmental Epidemiology Applied to Reproduction and Respiratory Health, Institute for Advanced Biosciences, Grenoble, France; Commissariat à l'Energie Atomique (CEA), IRIG department, INSERM U1292, and Grenoble Alpes University (UGA), Grenoble, France; Norwegian Institute of Public Health, Oslo, Norway; Norwegian Institute of Public Health, Oslo, Norway; Norwegian Institute of Public Health, Oslo, Norway; Department of Obstetrics/Gynecology and Fetal Medicine, Grenoble University Hospital, Grenoble, France; Université Paris-Saclay, UVSQ, INRAE, BREED, France; Ecole Nationale Vétérinaire d’Alfort, BREED, Maisons-Alfort, France; University Grenoble Alpes, Inserm U1209, CNRS UMR 5309, Team of Environmental Epidemiology Applied to Reproduction and Respiratory Health, Institute for Advanced Biosciences, Grenoble, France; University Grenoble Alpes, Inserm U1209, CNRS UMR 5309, Team of Environmental Epidemiology Applied to Reproduction and Respiratory Health, Institute for Advanced Biosciences, Grenoble, France

**Keywords:** placental weight, placental-to-fetal-weight ratio, phenols, phthalates, environmental exposures, mixture models, placental vascular resistance

## Abstract

**STUDY QUESTION:**

Is exposure to environmental chemicals associated with modifications of placental morphology and function?

**SUMMARY ANSWER:**

Phthalates, a class of ubiquitous chemicals, showed an association with altered placental weight, placental vascular resistance (PVR), and placental efficiency.

**WHAT IS KNOWN ALREADY:**

Only a few epidemiological studies have assessed the effects of phenols and phthalates on placental health. Their results were affected by exposure measurement errors linked to the rapid excretion of these compounds and the reliance on a limited number of spot urine samples to assess exposure.

**STUDY DESIGN, SIZE, DURATION:**

A prospective mother–child cohort, with improved exposure assessment for non-persistent chemicals, recruited participants between 2014 and 2017. Sample size ranged between 355 (placental parameters measured at birth: placental weight and placental-to-fetal weight ratio (PFR): a proxy for placental efficiency) and 426 (placental parameters measured during pregnancy: placental thickness and vascular resistance).

**PARTICIPANTS/MATERIALS, SETTING, METHODS:**

Phenols (four parabens, two bisphenols, triclosan, and benzophenone-3), 13 phthalate metabolites, and two non-phthalate plasticizer metabolites were measured in within-subject pools of repeated urine samples collected during the second and third trimesters of pregnancy (median = 21 samples/trimester/woman). Placental thickness and PVR were measured during pregnancy. The placenta was weighed at birth and the PFR was computed. Both adjusted linear regression and Bayesian Kernel Machine Regression were used to evaluate associations between phenols and phthalates (alone or as a mixture) and placental parameters. Effect modification by child sex was also investigated.

**MAIN RESULTS AND THE ROLE OF CHANCE:**

Several phthalate metabolites were negatively associated with placental outcomes. Monobenzyl phthalate (MBzP) concentrations, during the second and third trimesters of pregnancy, were associated with a decrease in both placental weight at birth (*β* = −20.1 g [95% CI: −37.8; −2.5] and *β* = −17.4 g [95% CI: −33.2; −1.6], for second and third trimester, respectively) and PFR (*β* = −0.5 [95% CI: −1, −0.1] and *β* = −0.5 [95% CI: −0.9, −0.1], for the second and third trimester, respectively). Additionally, MBzP was negatively associated with PVR during the third trimester (*β*= −0.9 [95% CI: −1.8; 0.1]). Mono-n-butyl phthalate (MnBP), was negatively associated with PVR in both trimesters (*β* = −1.3, 95% CI: [−2.3, −0.2], and *β* = −1.2, 95% CI: [−2.4, −0.03], for the second and third trimester, respectively). After stratification for child sex, Σ diisononyl phthalate (DiNP) (either second or third-trimester exposures, depending on the outcomes considered) was associated with decreased PVR in the third trimester, as well as decreased placental weight and PFR in males. No associations were observed for phenol biomarkers.

**LIMITATIONS, REASONS FOR CAUTION:**

False positives cannot be ruled out. Therefore, chemicals that were associated with multiple outcomes (MnBP and DiNP) or reported in existing literature as associated with placental outcomes (MBzP) should be considered as the main results.

**WIDER IMPLICATIONS OF THE FINDINGS:**

Our results are consistent with *in vitro* studies showing that phthalates target peroxisome proliferator-activated receptor γ, in the family of nuclear receptors involved in key placental development processes such as trophoblast proliferation, migration, and invasion. In addition to placental weight at birth, we studied placental parameters during pregnancy, which could provide a broader view of how environmental chemicals affect maternal–fetal exchanges over the course of pregnancy. Our findings contribute to the increasing evidence indicating adverse impacts of phthalate exposure on placental health.

**STUDY FUNDING/COMPETING INTEREST(S):**

This work was supported by the French Research Agency—ANR (MEMORI project ANR-21-CE34-0022). The SEPAGES cohort was supported by the European Research Council (N°311765-E-DOHaD), the European Community’s Seventh Framework Programme (FP7/2007-206—N°308333-892 HELIX), the European Union’s Horizon 2020 research and innovation programme (N° 874583 ATHLETE Project, N°825712 OBERON Project), the French Research Agency—ANR (PAPER project ANR-12-PDOC-0029-01, SHALCOH project ANR-14-CE21-0007, ANR-15-IDEX-02 and ANR-15-IDEX5, GUMME project ANR-18-CE36-005, ETAPE project ANR-18-CE36-0005—EDeN project ANR-19-CE36-0003-01), the French Agency for Food, Environmental and Occupational Health & Safety—ANSES (CNAP project EST-2016-121, PENDORE project EST-2016-121, HyPAxE project EST-2019/1/039, PENDALIRE project EST-2022-169), the Plan Cancer (Canc’Air project), the French Cancer Research Foundation Association de Recherche sur le Cancer—ARC, the French Endowment Fund AGIR for chronic diseases—APMC (projects PRENAPAR, LCI-FOT, DysCard), the French Endowment Fund for Respiratory Health, the French Fund—Fondation de France (CLIMATHES—00081169, SEPAGES 5–00099903, ELEMENTUM—00124527). N.J. was supported by a doctoral fellowship from the University Grenoble Alpes. V.M. was supported by a Sara Borrell postdoctoral research contract (CD22/00176), granted by Instituto de Salud Carlos III (Spain) and NextGenerationEU funds. The authors declare no conflict of interest.

**TRIAL REGISTRATION NUMBER:**

ClinicalTrials.gov NCT02852499.

WHAT DOES THIS MEAN FOR PATIENTS? The present study looked at whether exposure to phthalates and synthetic phenols can affect the health and growth of the placenta, a crucial organ for fetal development. These compounds are notorious for their widespread exposure because of their presence in numerous everyday products, such as food packaging, cosmetic products, and building materials. We relied on innovative methodologies to investigate the associations between phenols and phthalates (studied individually or as a mixture) and placental morphology and efficiency, assessed during pregnancy and at birth. Our results suggested that several phthalates, including butyl-benzyl phthalate (BBzP), and Di-n-butyl phthalate (DnBP) were associated with a decrease in placental weight and efficiency. Furthermore, our study indicated that some effects were modulated by the sex of the fetus, resulting in stronger associations for males. Given the key roles played by the placenta during the whole pregnancy, and the widespread exposure to phthalates, consequences for the fetus and mothers may be substantial.

## Introduction

The Developmental Origins of Health and Disease (DOHaD) paradigm emphasizes the crucial influence of early-life exposures on future health. The placenta, the main interface between the mother and the fetus, likely plays a pivotal role in child health programming. Changes in placental weight and placental-to-fetal weight ratio (PFR)—a proxy for placental efficiency—have indeed been associated with adverse health outcomes at birth (e.g. higher risk of cryptorchidism with lower placental weight (Arendt *et al.*, 2016), lower Apgar score (Bonds *et al.*, 1984), and increased risk of being small-for-gestational-age (Miwa *et al.*, 2014; Schwartz *et al.*, 2014)) and later in life such as increased risk of death owing to cardiovascular diseases (Risnes *et al.*, 2009).

Environmental factors, such as exposure to endocrine disrupting compounds, may affect the placenta, as demonstrated by toxicological studies (Zong *et al.*, 2015; Müller *et al.*, 2018; Blake *et al.*, 2020; Jiang *et al.*, 2020; Profita *et al.*, 2021). Among these are the synthetic phenol and phthalate families, to which the general population is ubiquitously exposed (Haug *et al.*, 2018; Rolland *et al.*, 2020; Philippat *et al.*, 2021). Two epidemiological studies relying on the same large cohort (n ∼ 2720), reported positive associations between several phthalate metabolites and placental thickness, breadth, length, and weight, and negative associations with the PFR (Zhu *et al.*, 2018; Gao *et al.*, 2022). Studies investigating phenol exposure, despite generally having smaller sample sizes (N between 91 and 488), successfully identified negative associations between triclosan (Ferguson *et al.*, 2018; Philippat *et al.*, 2019), benzophenone-3 (Philippat *et al.*, 2019), and placental weight. Interestingly, a contrasting result was observed for bisphenol A, wherein a positive association was reported (Casas *et al.*, 2016). Unfortunately, these studies were limited by the low number of urine samples used to assess exposure (from one (Philippat *et al.*, 2019) to three (Gao *et al.*, 2022)), which may be inadequate in capturing pregnancy exposure owing to the high temporal variability reported for some compounds (Casas *et al.*, 2018; Rolland *et al.*, 2020; Philippat *et al.*, 2021). This limitation could potentially lead to measurement errors and biased effect estimates towards the null (Perrier et al., 2016).

With one exception (Gao *et al.*, 2022), compounds were studied individually, even though pregnant women are simultaneously exposed to multiple phenols and phthalates that can share modes of action. Moreover, the effect of a few replacement compounds, such as bisphenol S and, 1,2-cyclohexane dicarboxylic acid diisononyl ester (DINCH) (replacements for bisphenol A and di(2-ethylhexyl) phthalate (DEHP) respectively), have not been studied despite data showing increased exposure over time (Gyllenhammar *et al.*, 2017; Kasper-Sonnenberg *et al.*, 2019; Frederiksen *et al.*, 2020) and toxicological studies suggesting that they may affect the placenta development (Gingrich *et al.*, 2018; Profita *et al.*, 2021).

We aimed to investigate the associations between exposure to phenols and phthalates (including bisphenol S and DINCH), individually and as a mixture, and placental health parameters measured both during pregnancy and at birth. We relied on multiple urine samples collected throughout pregnancy to enhance exposure assessment.

## Materials and methods

### Study population and recruitment

This study relied on a subsample of the French mother–child cohort SEPAGES (Lyon-Caen *et al.*, 2019), which recruited 484 pregnant women between July 2014 and July 2017. Inclusion criteria included: being older than 18 years old, being under 19 gestational weeks, singleton pregnancy, living in the study area (within 80 km of the center of Grenoble), and planning to deliver in one of the four maternity wards of Grenoble. The majority of the participants (90%) were recruited in ultrasound clinics.

Among the 484 included women, 479 provided urine samples during pregnancy, which were measured for phenol and phthalate metabolites. Among them, 426 had at least one ultrasound or Doppler measurement of placental thickness or placental vascular resistance (PVR) (second or third trimester), and for 355, information on placental weight at birth was available. A flowchart of the study population is displayed in Supplementary Fig. S1.

### Outcomes: placental morphometry and placental vascular resistance

Doppler ultrasound of the umbilical artery was performed in the second (N = 380) and third (N = 388) trimester of the pregnancy, allowing for the estimation of PVR, also known as the resistance index. PVR was expressed as a percentage and automatically computed from the systolic and diastolic umbilical blood flow by the Doppler instruments using the following formulae:

$$\mathrm{PVR}=\;\frac{\mathrm{Systolic}\;\mathrm{velocity}-\mathrm{Diastolic}\;\mathrm{velocity}}{\mathrm{Systolic}\;\mathrm{Velocity}}$$

An abnormal PVR often reflects atypical blood flow patterns in fetal circulation and can serve as an indicator of unfavorable fetal prognosis. Notably, a high PVR may prompt interventions such as early delivery (Rizzo *et al.*, 2021).

Placental thickness was measured by ultrasonography in the second trimester (N = 392).

At birth, the placenta was weighed by the medical staff. Birth weight was extracted from the medical record and used to compute the PFR, using the following formula:

$$\text{PFR}=\frac{\text{Placental}\;\text{weight}}{\mathrm{Birth}\;\mathrm{weight}}\;\times \;100$$

The PFR offers insights into placental efficiency in relation to fetal growth. A relatively small placenta compared to the fetus might suggest that it struggles to adequately support fetal development (Salavati *et al.*, 2019).

### Urine collection

Women were asked to collect three urine samples per day, over a period of 1 week. This process occurred twice during pregnancy (gestational age at the second and third trimester collection weeks (mean ± SD): 18 ± 2 and 34 ± 2 weeks, respectively). Samples were stored in the participants’ freezer until collection by a SEPAGES field worker. After collection, samples were transported on dry ice to a certified biobank (ISO-9001 standard, Grenoble University Hospital, bb-0033-00069). For each subject, weekly pools were created using an equal volume of all voids collected over each urine collection week. Research conducted by Philippat and Calafat in 2021 found that pooling urine samples collected over a week provided similar accuracy to the results obtained using methods which considered the volume or dilution of individual samples. Moreover, pooling allows for estimating biomarker concentrations in a way that is comparable to analyzing all the urine produced in that week (Philippat and Calafat, 2021).

### Exposures: urinary concentrations of phenol and phthalate metabolites

Aliquots of each available pool were stored at −80°C in the biobank, before being sent on dry ice to the Norwegian Institute for Public Health for measurement of eight phenols, 13 phthalate metabolites, and two non-phthalate plasticizer metabolites. Phthalates and DINCH biomarkers were analyzed using high-performance liquid chromatography coupled to mass spectrometry (HPLC MS/MS (Sabaredzovic *et al.*, 2015)). Phenols were measured using ultra-high-performance liquid chromatography coupled to mass spectrometry (UPLC MS/MS (Sakhi *et al.*, 2018)). Procedural blanks, in-house quality controls, and standard reference material (SRM 3673) from the National Institute of Standards and Technology were analyzed along with the samples in the aforementioned methods. Accuracy ranged from 70% to 126%, and the precision, given as relative SD, was below 26% for phenols, phthalates, and DINCH metabolites. The free and conjugated forms of phenol biomarkers were measured in samples from 50 women. Based on the preliminary measurements, external contamination was not detected (Rolland *et al.*, 2020). Therefore, for the remaining women in the study, we conducted analysis on the total form (free + conjugated).

### Statistical analysis

#### Urinary concentrations

When the detection rate was lower than 30%, urinary concentrations were dichotomized (below versus above the limit of detection (LOD)). For compounds with higher detection rates, we individually imputed values below LOD and between LOD and the limit of quantification (LOQ) by randomly selecting values randomly picked up between 0 and LOD, and LOD and LOQ, respectively, based on the estimated distribution of the metabolite (Lubin *et al.*, 2004).

After imputation, urinary concentrations were corrected for the following technical variables: transport time of the urine between participants’ house and biobank, and time spent unfrozen during pooling procedure and analytical batches. This step was performed if the above-mentioned conditions were associated with the concentration of the biomarker. To determine the need for correction, we first studied the associations between the conditions mentioned above and each biomarker concentration using adjusted linear regression models. We then used the effect estimate of each sampling condition associated with the given biomarker urinary concentration (*P*-value < 0.2) and the measured concentrations to predict the concentrations that would have been obtained if all women had their urine samples processed under the same conditions (Mortamais *et al.*, 2012; Guilbert *et al.*, 2021) (Supplementary Table S1).

The molar sums of all the metabolites that belonged to the same parent compound (DEHP, DINCH, and diisononyl phthalate (DiNP)) were computed (Supplementary Table S2).

#### Covariates selection, imputation, and coding

Confounders shown in the directed acyclic graph (DAG) were selected *a priori* (Supplementary Fig. S2). Variables identified as confounders or predictors of the outcome were included as adjustment factors in our final model, resulting in the following list of covariates: maternal parity (nulliparous vs uni/multiparous), maternal age at conception, a maternal education level (up to 2 years after high school/between 3 and 4 years/5 years or more), maternal active smoking during any trimester of pregnancy (No/Yes), maternal passive smoking during any trimester of pregnancy (exposed to <1 cigarette per week/exposed to one cigarette per week or more), maternal pre-pregnancy BMI, gestational age at birth or at the ultrasound measurement depending on the outcomes and infant’s sex (female/male). We also adjusted for place of delivery for outcomes measured at birth (i.e. placental weight and PFR).

Missing values for covariates were generally low (<10%, Supplementary Table S3) and we singly imputed them using the median in the study population (continuous variables) or the most prevalent level (categorical variables).

#### Adjusted association with placental parameters: single-chemical models

We first constructed one model per exposure/outcome pair, encompassing all combinations between our exposures and five outcomes (two in the second trimester, one in the third trimester, and two at birth). Models were adjusted on the relevant covariates based on the timing of the outcome.

Owing to the reporting of non-linear associations (Gao *et al.*, 2022), each phenol and phthalate concentration was first broken down into terciles, before being placed in an adjusted regression model against placental weight.

This allowed us to compute the *P*-value for heterogeneity between the terciles with Wald’s test. To estimate a *P*-trend, representing a trend across all the terciles, the median of each terciles was taken and used in the regression models against placental weight. A low Wald’s test *P*-value and a high *P*-trend was considered as a non-linear response. For compounds that did not suggest a non-linear relationship in our above-mentioned analysis, we relied on the continuous biomarker concentrations (ln-transformed) in our model.

Since previous studies have reported a possible effect modification by the infant’s sex (Matsuda *et al.*, 2015; Casas *et al.*, 2016; Ogawa *et al.*, 2016), an interaction term between biomarker urinary concentration and the infant’s sex was added in the third set of models. For interaction, *P*-values equal to or lower than 0.1, an additional sex stratification analysis was performed.

#### Additional sensitivity analysis (single-chemical models)

To evaluate the robustness of our single-chemical analysis, several sensitivity analyses were performed.

Women with missing placental weight at birth were observed to have a higher likelihood of delivering in maternity ward 1 and undergoing a C-section (Supplementary Table S3). Hence, to mitigate potential selection bias, we conducted an additional analysis, using inverse probability weighting (IPW) (Hernán and Robins, 2020). For each participant, we assigned a weight equal to the inverse of the probability of having a placental weight, and thus of being integrated in the main analysis.

To compute this weight, we ran a logistic regression model to predict the probability that a woman had a placental weight measurement. The covariates included in this model were all the main model’s covariates, plus the mode of delivery. The inverse of this probability was then utilized as a weight for the individual chemical models investigating associations with placental weight and PFR.

We evaluated the impact of influential values by estimating studentized residuals and excluding participants whose residual values were outside the −3, 3 range (N of excluded values ranged between 0 and 5 depending on the exposure–outcome pair).

A previous study suggested that, for bisphenol A, accounting for urine dilution when using equal volume pools was not suitable (Philippat and Calafat, 2021), hence biomarker urinary concentrations were not corrected for specific gravity in our main analysis. However, since the aforementioned study focused on only two substances (bisphenol A and triclosan), we performed an additional sensitivity analysis using biomarker concentration corrected for specific gravity, a marker of urine dilution.

Finally, because gestational age at birth could be on the causal pathway (DAG, Supplementary Fig. S2), we also ran new models without gestational age as a covariate.

#### Adjusted association with placental parameters: mixture models

We used Bayesian Kernel Model Regression (BKMR, R package: {bkmr}, (Bobb *et al.*, 2015)) to assess the effect of the chemical mixtures on placental outcomes. Chemicals were grouped per trimester and each model was run for 50,000 iterations. BKMR only accepts numerical variables, thereby bisphenol S and butyl paraben were not considered for this analysis. Results were evaluated graphically. We plotted the expected change in each placental outcome with a concomitant increase in quantiles of all exposure biomarkers included in the mixture, relative to when they are fixed at the 10th percentile. This analysis was performed in the whole population as well as after stratification for child sex.

We established statistical significance at a *P*-value below 0.05. A *P*-value ranging from 0.05 to 0.10 was viewed as potentially indicative of a relationship if it followed a pattern of associations (i.e. a non-isolated association). For the discussion and conclusion sections, we have only considered chemicals that were either associated with several outcomes and/or have shown an association with placental outcomes in the existing literature.

Analyses were performed using R 4.2.1 (R Foundation, Vienna, Austria). Packages used included targets for analysis pipeline management, dplyr for dataframe manipulation, future for parallelization, tibble for dataframe manipulation, gtsummary for publication-ready table production, corrplot for correlation plots, mice for handling multiple imputations, stringr for strings (character chains) manipulation, bkmr for running BKMR model and tidymodels for model creation. The code is available at https://gricad-gitlab.univ-grenoble-alpes.fr/iab-env-epi/jovanovic_exploring_2023.

### Ethics approval

Ethical agreements were obtained from the CPP (Comité de Protection des Personnes Sud-Est V) and the Commission Nationale de l'Informatique et des Libertés (CNIL), the French data privacy institution. All participants provided written informed consent before enrollment.

## Results

### Study population description

Most of the participants had a high level of education, with 56% having a Master’s degree or higher (Supplementary Table S3). Tobacco exposure was limited, with 93% non-smokers and 81% not exposed to passive smoke during the pregnancy. The mean age at conception was 32 years, with the majority (55%) of participants having already given birth to a child. The male-to-female ratio at birth was 1.13. The median of gestation duration was at 40 weeks. Mean placental weight and birthweight were 533 g and 3,309 g, respectively (Supplementary Table S4). Spearman correlation coefficients between placental outcomes were low (≤ 0.15, Supplementary Fig. S3), except between placental weight and PFR, since the former was used to calculate the latter (rho = 0.74).

### Exposure assessment

The median number of urine samples per urine collection week was 21 (25^th^ percentile: 20; 75^th^ percentile: 21).

Bisphenols F, B, AF, and triclocarban were detected in <2% of the pooled urine samples and were not considered in the statistical analyses. Butyl paraben and bisphenol S detection rates were < 30% regardless of the pregnancy period and thus were dichotomized. Except for propyl paraben, which had a detection rate between 78% and 79%, all other metabolites were detected in at least 98% of the samples (Table 1).

**Table 1.** Standardized urinary concentrations of phenols and phthalates among pregnant women of the ultrasound group.

			**Second trimester** (N = 469)					**Third trimester** (N = 469)				
Chemical	LOD	LOQ	%>LOD	%>LOQ	33th	Median	66th	%>LOD	%>LOQ	33th	Median	66th
Bisphenol A	0.04	0.1	99.6	99.4	1.4	1.84	2.5	98.4	98	1.22	1.67	2.31
Bisphenol S	0.1	0.4	24.4	20.6	—	—	—	27.7	22.1	—	—	—
Methyl paraben	0.04	0.1	100	100	6.7	11.2	22.97	100	100	6.73	11.58	24.76
Ethyl paraben	0.04	0.1	99.8	99.8	0.6	0.89	1.32	99.8	99.3	0.61	0.92	1.37
Propyl paraben	0.04	0.1	79.2	67.9	0.1	0.34	1.55	78.1	66.5	0.09	0.44	1.9
Butyl paraben	0.07	0.2	24.6	10.7	—	—	—	23.9	12.1	—	—	—
Triclosan	0.04	0.1	98.5	98.5	0.6	0.92	1.57	98.2	98	0.57	0.87	1.37
Benzophenone-3	0.04	0.1	100	98.7	0.5	0.83	1.47	99.8	98.9	0.47	0.72	1.21
MEP	0.2	0.5	100	100	14.9	23.91	36.45	100	100	12.91	20.93	30.96
OH-MPHP	0.07	0.2	100	99.6	0.8	0.86	1.02	100	99.6	0.7	0.82	0.94
MBzP	0.07	0.2	100	100	3.50	4.44	5.76	100	100	3.19	4.14	5.6
MiBP	0.2	0.5	100	100	12.2	14.79	19.59	100	100	11.29	14.6	18.88
MnBP	0.2	0.5	100	100	8.72	10.55	13.52	100	100	8.66	11.25	13.92
MEHP	0.2	0.5	100	99.1	1.74	2.35	3.22	99.8	94.6	1.33	1.92	2.55
MEHHP	0.2	0.5	100	100	5.66	6.94	9.04	100	100	5.41	7.11	8.97
MEOHP	0.2	0.5	100	100	3.99	4.89	6.67	100	100	4.03	5.27	6.52
MECPP	0.7	2	100	99.8	8.42	9.78	12.15	100	100	8.05	10.01	12.38
MMCHP	0.7	2	99.4	99.1	6.27	7.47	9.27	99.6	99.6	6.27	7.53	9.13
∑DEHP	—	—	—	—	0.09	0.11	0.13	—	—	0.09	0.11	0.13
oxo-MINCH	0.07	0.2	100	99.8	1.15	1.5	1.95	100	99.8	1.11	1.53	2.06
oh-MINCH	0.07	0.2	100	100	1.32	1.73	2.38	100	100	1.2	1.63	2.14
∑DINCH	—	—	—	—	0.01	0.01	0.01	—	—	0.01	0.01	0.01
oxo-MiNP	0.1	0.25	100	99.6	1.6	2.18	2.83	100	100	1.55	2.01	2.73
oh-MiNP	0.1	0.25	100	100	3.44	4.94	7.81	100	100	3.12	4.48	6.63
cx-MiNP	0.4	1	100	100	3.89	4.68	5.89	100	100	3.68	4.46	5.34
∑DiNP	—	—	—	—	0.03	0.04	0.06	—	—	0.03	0.04	0.05

Data are µg/l or µmol/l for molar sum. Bisphenols F-B-AF and triclocarban are not displayed here because they were detected in <2% of the pooled urine samples.

∑, molar sum; MEP, monoethyl phthalate; MnBP, mono-n-butyl phthalate; MiBP, mono-isobutyl phthalate; MBzP, monobenzyl phthalate; OH-MPHP, 6-hydroxy-mono-propyl-heptyl phthalate; MEHP, mono(2-ethylhexyl) phthalate; MEHHP, mono(2-ethyl-5-hydroxyhexyl) phthalate; MEOHP, mono(2-ethyl-5oxohexyl) phthalate; MECPP, mono(2-ethyl-5-carboxypentyl) phthalate; MMCHP, mono-2-carboxymethyl hexyl phthalate; oxo-MiNP, mono-4-methyl-7-oxooctyl phthalate; OH-MiNP, mono-4-methyl-7-hydroxyoctyl phthalate; cx-MiNP, mono-4-methyl-7-carboxyoctylphthalate; oxo-MINCH, 2-(((4-Methyl-7oxyooctyl)oxy)carbonyl)cyclohexanecarboxylicacid; OH-MINCH, 2-(((Hydroxy-4-methyloctyl)oxy)carbonyl)cyclohexanecarboxylic acid; DINCH, di(isononyl)cyclohexane-1,2-dicarboxylate; DiNP, diisononyl phthalate; DEHP, di(2-ethylhexyl) phthalate; LOQ, limit of quantification; LOD, limit of detection.

### Adjusted associations with placental health: single-chemical models

Our analysis relying on exposure coded in terciles did not highlight major deviations from the linearity for most chemicals (Supplementary Table S5), and thus biomarker concentrations were evaluated as continuous variables in subsequent models.

### Phthalates

**Figure 1.**
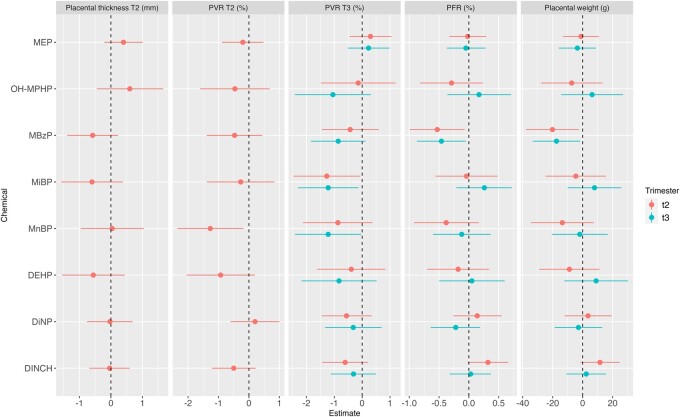
**Adjusted associations between phthalate urinary biomarkers and placental parameters measured at birth and during pregnancy.** MBzP, monobenzyl phthalate; MEP, monoethyl phthalate; MnBP, mono-n-butyl phthalate; MiBP, mono-isobutyl phthalate; OH-MPHP: 6-hydroxy-mono-propyl-heptyl phthalate; DEHP: di(2-ethylhexyl) phthalate; DiNP: diisononyl phthalate; 1,2-cyclohexane dicarboxylic acid diisononyl ester; T2: second trimester; T3: third trimester; PFR: placental to foetal ratio; PVR, placental vascular resistance. Adjustment factors: maternal age, parity, education level, active smoking, passive smoking, BMI before pregnancy, gestational duration, maternity ward. Placental thickness T2: N = 392; PVR T2: N = 380; PVR T3: N = 388; PFR and placental weight: N = 355.

**Figure 2.**
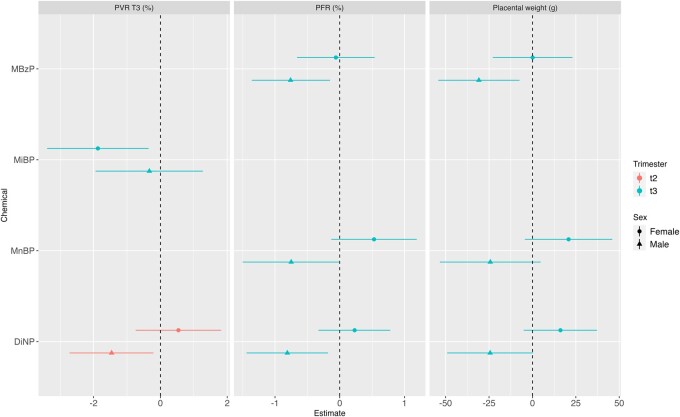
**Analysis stratified by infant’s sex: adjusted associations between phthalate urinary biomarkers and placental parameters measured at birth and during pregnancy.** MnBP: mono-n-butyl phthalate; MiBP: mono-isobutyl phthalate; MBzP: monobenzyl phthalate; DiNP: diisononyl phthalate; 1,2-cyclohexane dicarboxylic acid diisononyl ester; PVR, placental vascular resistance; PFR, placental-to-foetal-weight ratio; T3: third trimester. Only associations for which there was a suggestion for an interaction (P-value for interaction ≤ 0.1) are displayed.

Several phthalate metabolites were negatively associated with placental outcomes. Every unit increase in the ln-transformed monobenzyl phthalate (MBzP) urinary concentration was associated with a reduction in placental weight (*β* = −20.1 g [95% CI: −37.8; −2.5] and *β* = −17.4 g [95% CI: −33.2; −1.6] for exposure in the second and third trimester, respectively) and PFR at birth (*β* = −0.5% [95% CI: −1, −0.1] and *β* = −0.5% [95% CI: −0.9, −0.1] for second and third trimester, respectively). MBzP in the third trimester also tended to be associated with a decrease in PVR measured in the third trimester (*β* = −0.9% [95% CI: −1.8; 0.1]) (Fig. 1, Supplementary Table S6). Since the *P*-value for interaction was 0.1, sex stratification was performed, revealing negative associations with placental and PFR for males only (N = 190 males, Fig. 2, Supplementary Table S7).

MnBP was associated with a reduction of PVR in both second (*β* = −1.3%, 95% CI: [−2.4, −0.2]) and third trimesters (*β* = −1.2% [95% CI: −2.4, −0.03]). After stratification for child sex, MnBP in the third trimester also tended to be negatively associated with PFR and placental weight in males but positively in females (Fig. 2).

MiBP exposure in both trimesters was associated with a decrease in PVR in the third trimester (*β* = −1.3% [95% CI: −2.5, −0.1] and *β* = −1.2% [95% CI: −2.3, −0.2], for exposure in the second and third trimester, respectively). Interaction with child sex was seen and this negative association persisted for the third trimester in females alone (Fig. 2 and Supplementary Table S7).

We were unable to capture any significant associations between the remaining phthalates metabolized and placental parameters in the studied population. However, effect modification by child sex was suggested for ΣDiNP (interaction *P*-value = 0.1). After stratification, this compound (either second or third trimester exposures, depending on the outcomes) was associated with a decreased PVR in the third trimester, placental weight, and PFR in males, but not in females (Fig. 2 and Supplementary Table S7).

### Phenols

**Figure 3.**
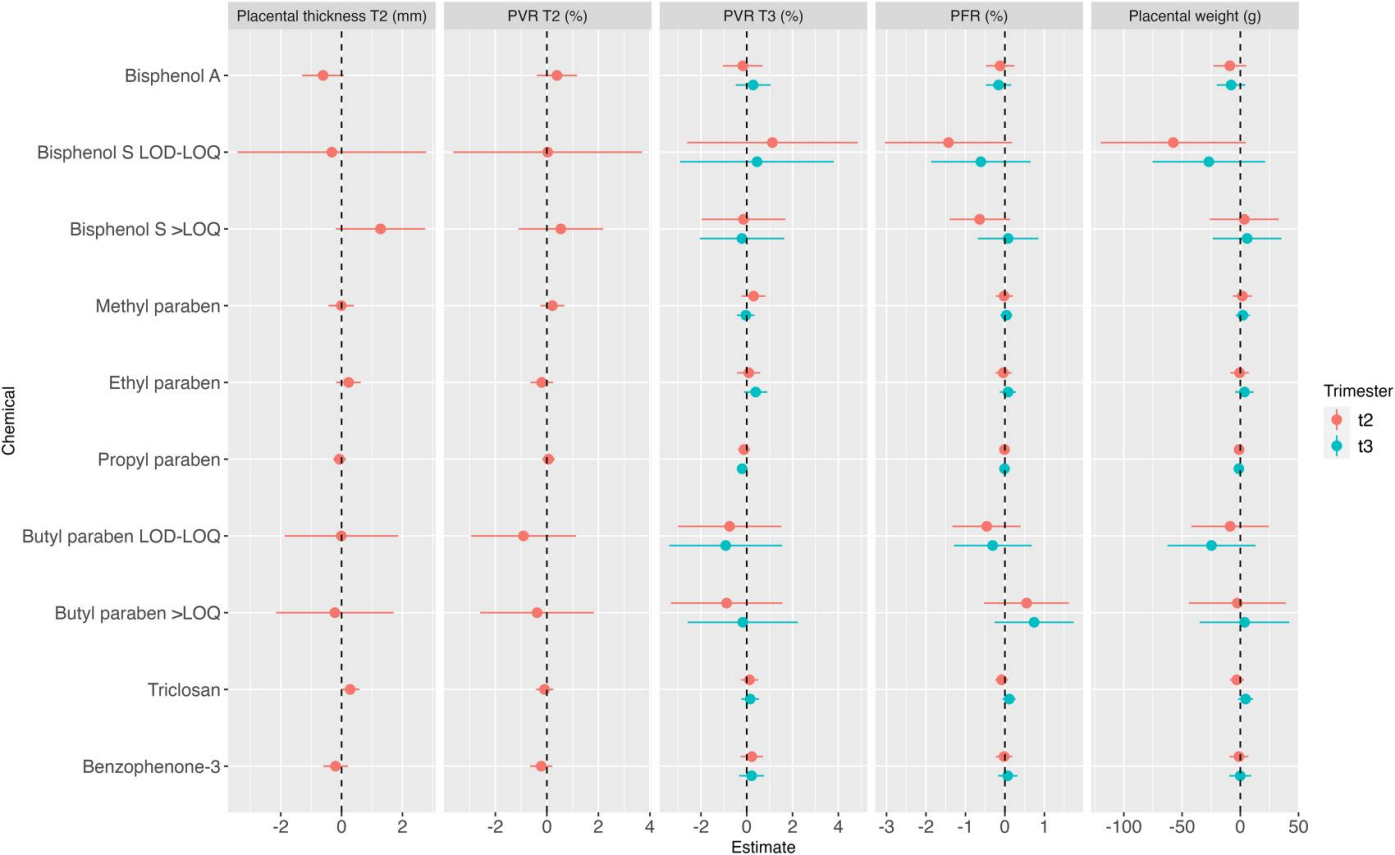
**Adjusted associations between phenol urinary biomarkers and placental parameters measured at birth and during pregnancy.** PFR, placental-to-foetal-weight ratio; PVR, placental vascular resistance; T2, second trimester; T3, third trimester. Adjustment factors: maternal age, parity, education level, active smoking, passive smoking, BMI before pregnancy, gestational duration, maternity ward. Placental thickness T2: N = 392; PVR T2: N = 380; PVR T3: N = 388; PFR and placental weight: N = 355.

**Figure 4.**
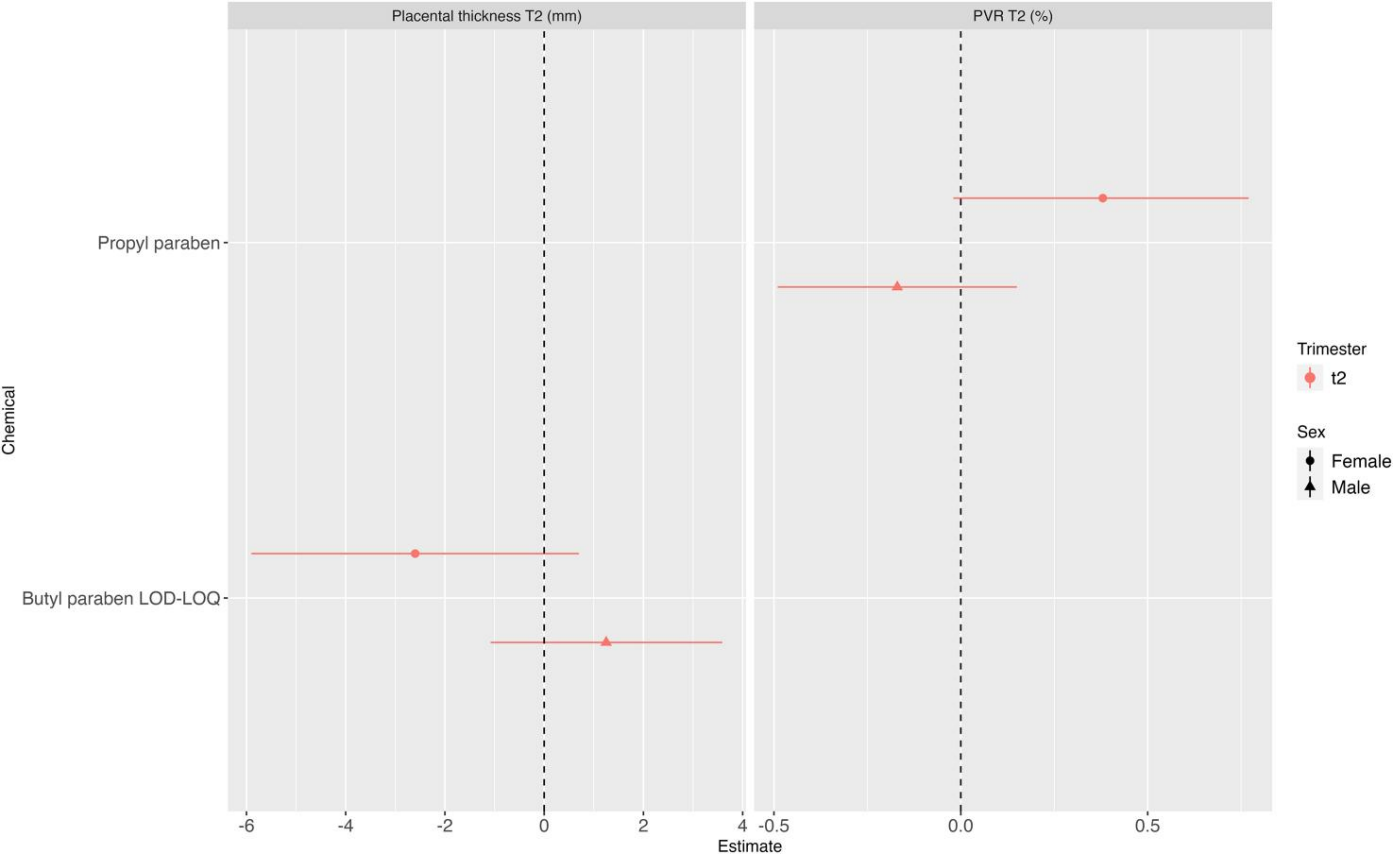
**Analysis stratified by infant’s sex: adjusted associations between phenol urinary biomarkers and placental parameters measured at birth and during pregnancy.** PVR: placental vascular resistance; T2, second trimester. Only associations for which there was a suggestion for an interaction (P-value for interaction ≤ 0.1) are displayed.

We did not observe any association between phenol biomarkers and placental parameters (Figs. 3 and 4 and Supplementary Table S6).

### Sensitivity analysis: single-chemical models

Overall, our sensitivity analysis mirrored the insights gleaned from the main analysis.

When IPW was applied to account for potential selection bias, the associations observed in our main analysis with PFR and placental weight were preserved (Supplementary Table S8). The sex-specific association between ΣDiNP in the third trimester and placental weight in males (*β* [95% CI] before/after: −24.4% [−49.1; 0.3]/−20% [−44.4; 4.4]), and MnBP in the third trimester and PFR (*β* [95% CI] before/after: −0.8% [−1.5; −0.01]/−0.7% [−1.4; 0.1]) in males showed slight attenuation.

After exclusion of influential values identified from studentized residuals (3–5 individuals depending of the outcome-exposure pairs) the negative association observed between MBzP in the third trimester and placental weight and PFR at birth were slightly attenuated (*β* for placental weight and PFR were −0.5% [95% CI −0.9: −0.1] and −17.4% [95% CI −33.2; −1.6] after their exclusion compared to −11.5% [95% CI: −26.8; 3.8] and −0.3% [95% CI: (−0.6; 0.1]) (Supplementary Table S9).

When specific gravity was added as a technical variable for the correction of exposure variables, all MBzP’s associations with PFR and placental weight were reduced by a small percentage, ranging from −5% to −22%. The most substantial change occurred with MBzP in the second trimester and PFR (*β* (95% CI) in the main model versus the sg-corrected one: −0.5 (−1; −0.1) versus −0.4 (−0.9; 0.1)) (Supplementary Table S10).

No substantial changes were observed when gestational age was removed from the list of covariates (Supplementary Table S11).

### Mixture analysis (BKMR models)

Even though the BKMR models did not highlight any significant associations with the mixture (Supplementary Figs S4, S5, S6, S7, S8, S9, S10, S11, S12, S13), a downward trend was observed mainly for PVR in the third trimester and PFR, both in males (Supplementary Figs S9, S11). Post-integration probabilities are available in Supplementary Table S12.

## Discussion

The aim of the present study was to determine whether exposure during pregnancy to phenols and phthalates, individually or in a mixture, had an impact on placental health. Repeated urine samples were collected to assess exposure. Our results revealed negative associations between individual phthalate metabolites, namely MnBP, MBzP, and ΣDiNP (in males only) and placental weight, PFR and PVR. When an interaction with child sex was detected, effects were overall stronger for male neonates. We did not observe any association with phenols, regardless of whether the exposure was studied individually or as part of a mixture (BKMR analysis).

The conclusions drawn from our study are intended for population-level considerations and should not be extrapolated to clinical implications. While applying these findings to global child health is inherently complex, it is nevertheless crucial to recognize the widespread nature of phthalate exposure and acknowledge that even slight variations in placental and birth outcomes across the entire population, influenced by contaminants, could lead to significant changes at the extremes of the distribution. This, in turn, might result in an increased number of cases with low birthweight and pregnancy complications.

Additionally, direct comparisons of our findings with past literature on the link between placental parameters and adverse health outcomes may be challenging. This complexity arises from use of continuous parameters in our study in contrast to the categorical approach used in previous research (Moore *et al.*, 1999; Shehata *et al.*, 2011; Miwa *et al.*, 2014; Arendt *et al.*, 2016).

### Association between phenols and placental parameters

We did not observe any association between the phenol biomarkers and placental health.

This is concurrent with findings in the literature (Casas *et al.*, 2016; Ferguson *et al.*, 2018). Contrastingly, associations with parabens (molar sum or ethyl paraben) have been reported (Philippat *et al.*, 2019; Vrijens *et al.*, 2020). Contradictory results have been reported, wherein the first study (restricted to male newborns) reported positive associations with placental weight (Philippat *et al.*, 2019), while the second one reported a negative association (Vrijens *et al.*, 2020). Noteworthy is the difference in the measurement of paraben concentration. In the second study, it was measured in the placenta, in contrast to the first study where urine was used as the medium. The paraben concentrations measured in our cohort—median urinary ethyl paraben concentration 0.7 µg/l—were significantly lower than those reported by Philippat *et al.* (2019) (3.11 µg/l). This disparity could potentially account for the non-significant association observed.

### Phthalates and placental parameters

According to our results, exposure to MBzP in both trimesters tended to decrease placental weight and PFR at birth, especially for male neonates. This finding has potential impact on fetal health, as low placental weight has been associated with increased umbilical artery pulsatility index at 20 weeks of gestation (WG), a marker of reduced placental function and slower growth of fetal abdominal circumference between 20 and 36 WG (Salavati *et al.*, 2016). Although a low PFR could indicate good placental efficiency, it could also reflect a placenta working at maximum capacity for its size (Salavati *et al.*, 2018). A low PFR has been associated with adverse perinatal outcomes, such as low Apgar scores and hyperbilirubinemia (Bonds *et al.*, 1984). The link between MBzP and placental parameters has also been investigated extensively (Casas *et al.*, 2016; Zhu *et al.*, 2018; Mustieles *et al.*, 2019; Philippat *et al.*, 2019). Casas *et al.* (2016) found increased placental weight and PFR in males, contrary to our findings. Interestingly, the other studies did not report any associations for MBzP (Zhu *et al.*, 2018; Mustieles *et al.*, 2019; Philippat *et al.*, 2019). Notably, these studies either did not explore sex-specific associations (Mustieles *et al.*, 2019; Philippat *et al.*, 2019), relied on a single urine sample (Philippat *et al.*, 2019), or assessed BBzP (the parent compound of MBzP) in cord blood, a matrix not preferred for chemicals characterized by a short half-life. (Zhu *et al.*, 2018).

We found MnBP and ΣDiNP (only in males) to be negatively associated with PVR, suggesting greater exchanges between the mother and the child. Although this is the first study investigating PVR in association with phthalates exposure, several other studies looked at maternal blood pressure, which may be somewhat associated with PVR. One study found an association between some phthalate metabolites (mainly MEP and MiBP) and decreased blood pressure in pregnant women (Warembourg *et al.*, 2019), whereas another (Bedell *et al.*, 2021) reported an increase in blood pressure differences between the third and the first trimester with increased exposure to MEP, MBP, and DEHP. Furthermore, a third study found no association (Han *et al.*, 2020).

For a few phthalate metabolites (MBzP, MnBP, ΣDiNP), a differential influence was observed between male and female children, suggesting potential interactions with child sex. After stratification, all of these associations were detected for male neonates. Given that the mechanisms behind such sex-specific effects are unknown, and because of our relatively small sample size, these results should be interpreted with caution. However, similar sex-specific associations were reported for a larger cohort for which a sex-stratified analysis was also conducted (Zhu *et al.*, 2018).

### Underlying biological pathways

Mechanisms by which phthalates may affect placental health include peroxisome proliferator-activated receptor γ (PPARγ), a nuclear receptor targeted by phthalates and involved in key placental development processes such as trophoblast proliferation, migration, and invasion (Fournier *et al.*, 2007). This receptor is also known to be involved in vascular tone, and thus blood pressure regulation (Kvandová *et al.*, 2016). Importantly, PPARγ is abundantly expressed in the cyto and syncytiotrophoblast cells of the human placenta. It may be involved in the transformation of the maternal spiral arteries from high- to low-resistance vessels that result from the invasion of extravillous trophoblasts cells in early pregnancy (Garnier *et al.*, 2015; Kvandová *et al.*, 2016). Additionally, a previous epidemiological study has shown associations between phthalate metabolites and placental angiogenic markers measured in maternal blood (Ferguson *et al.*, 2015). Specifically, DEHP was found to be associated with a reduction in the concentration of the placental growth factor, a member of the vascular endothelial growth factor family, that plays a crucial role in trophoblast invasion and placental vascularization (Alfaidy *et al.*, 2014).

### Implications for placental and fetal health

Among the outcomes studied in our article, only PVR can be seen as a clinical outcome (Rizzo *et al.*, 2021). However, we observed decreased PVR with increased exposure to phthalates, which was unexpected since low PVR may reflect improved fetal circulation and thus greater exchanges between the mother and the child.

While these are not standard clinical measurements, we propose that placental weight and PFR, alongside other placental parameters, may serve as an archive of the pregnancy, offering insights into how the prenatal environment, including exposure to chemicals, might affect child health. Our proposition is supported by various studies reporting associations between these parameters and health outcomes, be it at birth (Bonds *et al.*, 1984; Miwa *et al.*, 2014; Schwartz *et al.*, 2014; Arendt *et al.*, 2016) or later in life (Risnes *et al.*, 2009).

While the effects we observed may not be notable for individuals, the widespread nature of phthalate exposure means even minor variations can, across a population, lead to significant shifts at the tails of the distribution curve, and thus result in increased proportion of low birthweight babies and pregnancy complications.

### Strengths and limitations

To assess exposure during pregnancy, we analyzed repeated urine samples (two pools of up to 21 samples per woman each). If these repeated urine samples are collected during sensitive time windows, this should minimize measurement error, as well as any resulting bias in effect estimates compared to previous studies with similar sample size, but relying on a limited number of urine samples (Perrier et al., 2016). Furthermore, we corrected urinary concentrations based on technical variables, such as analytical batch, thus limiting associated variability. We investigated the effects of under-studied chemicals such as DINCH and bisphenol S. However, the low frequency of detection for bisphenol S (below 26%) may have limited our ability to detect an association for this compound. Further epidemiological studies will be needed to investigate the potential effects of these compounds on placental health.

Regarding the evaluation of mixture effects, we found only one other study, restricted to phthalates, that also investigated this type of effect (Gao *et al.*, 2022).

Owing to the exploratory nature of our analysis and the fact that most of the compounds studied have previously been associated with either placental or fetal health outcomes, we did not formally correct our analysis for multiple comparisons, and hence cannot rule out the possibility of false positives. This was the driving force behind focusing our discussion on chemicals for which a pattern was observed (chemicals associations with several outcomes) and/or for which an association with a placental parameter has been previously reported. Other isolated associations should be considered exploratory and warrant replication.

Placental weight at birth was missing for 26% of the study population. As this could have led to a selection bias, we performed a sensitivity analysis by using IPW. This analysis produced similar results to the main model. In addition to placental weight, we also relied on ultrasound and Doppler to measure placental thickness and PVR, respectively, allowing for the longitudinal monitoring of placental development over the course of the pregnancy.

In our study, these measurements were conducted by medical personnel, including sonographers and midwives, as part of routine antenatal care. This contrasts with a scenario where measurements would be consistently performed by the same healthcare professional, thus reducing variability. This variation may have potentially limited our ability to detect certain associations for these placental outcomes. The lack of correlation we observed between placental parameters aligns with findings from previous research (Afrakhteh *et al.*, 2013) and may have resulted from the time elapsed between these measurements (on average, there was a time gap of 10 weeks between the two Doppler measurements in SEPAGES). Additionally, the variations in certain parameters that naturally occur over the course of pregnancy, such as the usual decrease in PVR as gestational age increases (Acharya *et al.*, 2005; Krishna and Bhalerao, 2011)), could also explain the observed lack of correlation. We recommend future studies to include additional placental parameters, such as uterine Doppler, placental diameter, shape, and vascularization. This could potentially provide a broader view of how environmental chemicals affect placental health and maternal-fetal exchanges over the course of pregnancy. As an example, Doppler of the uterine artery along with notch diagnosis would provide a broader picture of the potential effects of phthalate on materno-fetal exchanges.

## Conclusion

Relying on improved exposure assessment and markers of placental health and functioning over the course of pregnancy, our study results suggest negative associations between individual phthalate metabolites and placental weight (MBzP and ΣDiNP), PFR (MBzP, MnBP and ΣDiNP), and PVR (MBzP, MnBP and ΣDiNP). These results lend further weight, alongside the existing literature, to the hypothesis that exposure to phthalates is associated with altered placental health and functioning. Additional research is recommended to assess the impact of these changes on fetal health.

## Supplementary Material

hoae018_Supplementary_Data

## Data Availability

Data used in this study are confidential and can only be provided upon a reasonable request to the SEPAGES steering committee. The code is available under the link: https://gricad-gitlab.univ-grenoble-alpes.fr/iab-env-epi/jovanovic_exploring_2023.
