# Supplementary material for: Associations between synthetic phenols, phthalates, and placental growth/function: a longitudinal cohort with exposure assessment in early pregnancy
Source: Hum Reprod Open. 2024 Apr 1;2024(2):hoae018. doi: 10.1093/hropen/hoae018 (PMC11057944; doi:10.1093/hropen/hoae018)
Supplement: hoae018_Supplementary_Data [file hoae018_supplementary_data.docx]

**Associations Between Synthetic Phenols, Phthalates, and Placental Growth / Function: A Longitudinal Cohort with Exposure Assessment in Early Pregnancy**

Authors: N. Jovanovic, V. Mustieles, M. Althuser, S. Lyon-Caen, N. Alfaidy, C. Thomsen, A.K. Sakhi, A. Sabaredzovic, S. Bayat, A. Couturier-Tarrade, R. Slama^1^, C. Philippat

**Table of contents**

Supplementary Figures

[Supplementary Figure S1 – Flowchart of the study population selection](#_Toc162027947)

[Supplementary Figure S2 - Directional Acyclic Graph for placental weight](#_Toc162027948)

[Supplementary Figure S3 – Outcomes correlation plot](#_Toc162027949)

[Supplementary Figure S4 – BKMR Analysis: Quantile-based mixture effect on placental thickness at the second trimester.](#_Toc162027950)

[Supplementary Figure S5 – BKMR Analysis: Quantile-based mixture effect on placental thickness at the second trimester with sex-stratified population.](#_Toc162027951)

[Supplementary Figure S6 – BKMR Analysis: Quantile-based mixture effect on placental vascular resistance at the second trimester.](#_Toc162027952)

[Supplementary Figure S7 – BKMR Analysis: Quantile-based mixture effect on placental vascular resistance at the second trimester with sex-stratified population.](#_Toc162027953)

[Supplementary Figure S8 – BKMR Analysis: Quantile-based mixture effect on placental vascular resistance at the third trimester.](#_Toc162027954)

[Supplementary Figure S9 – BKMR Analysis: Quantile-based mixture effect on placental vascular resistance at the third trimester with sex-stratified population.](#_Toc162027955)

[Supplementary Figure S10 – BKMR Analysis: Quantile-based mixture effect on PFR.](#_Toc162027956)

[Supplementary Figure S11 – BKMR Analysis: Quantile-based mixture effect on PFR with sex-stratified population.](#_Toc162027957)

[Supplementary Figure S12 – BKMR Analysis: Quantile-based mixture effect on placental weight.](#_Toc162027958)

[Supplementary Figure S13 – BKMR Analysis: Quantile-based mixture effect on placental weight with sex-stratified population.](#_Toc162027959)

[Supplementary tables](#_Toc162027960)

[Supplementary Table S1 – Standardization parameters for each exposure variable](#_Toc162027961)

[Supplementary Table S2 – Molecular weights used in molar sum computation](#_Toc162027962)

[1,2-Cyclohexane dicarboxylic acid diisononyl ester (DINCH)](#_Toc162027963)

[Supplementary Table S3 – Population characteristics between women with and without placental weight measurement available at birth](#_Toc162027964)

[Supplementary Table S4 – Distribution of the placental parameter assessed during pregnancy and at birth](#_Toc162027965)

[Supplementary Table S5 – Adjusted associations between urinary biomarkers in terciles and placental weight](#_Toc162027966)

[Supplementary Table S6 – Adjusted associations between standardized phenols and phthalates urinary concentrations and placental outcomes in the whole population.](#_Toc162027967)

[Supplementary Table S7 – Adjusted associations between standardized phenols and phthalates urinary concentrations and placental outcomes in sex-stratified population.](#_Toc162027968)

[Supplementary Table S8 –Sensitivity analysis: Adjusted associations between standardized phenols and phthalate metabolites urinary concentrations and PFR and placental weight – weighted analysis^a^](#_Toc162027969)

[Supplementary Table S9 -Sensitivity analysis, removal of influential values: Adjusted associations between standardized phenols and phthalates urinary concentrations and placental outcomes](#_Toc162027970)

[Supplementary Table S10 – Sensitivity analysis: Adjusted associations between standardized phenols and phthalates urinary concentrations and placental outcomes, analysis additionally adjusted on specific gravity](#_Toc162027971)

[Supplementary Table S11 - Adjusted associations between standardized phenols and phthalates urinary concentrations and placental outcomes, not adjusted on gestational age.](#_Toc162027972)

[Supplementary Table S12 – BKMR Models’ Post-Integration Probabilities (PIPs)](#_Toc162027973)


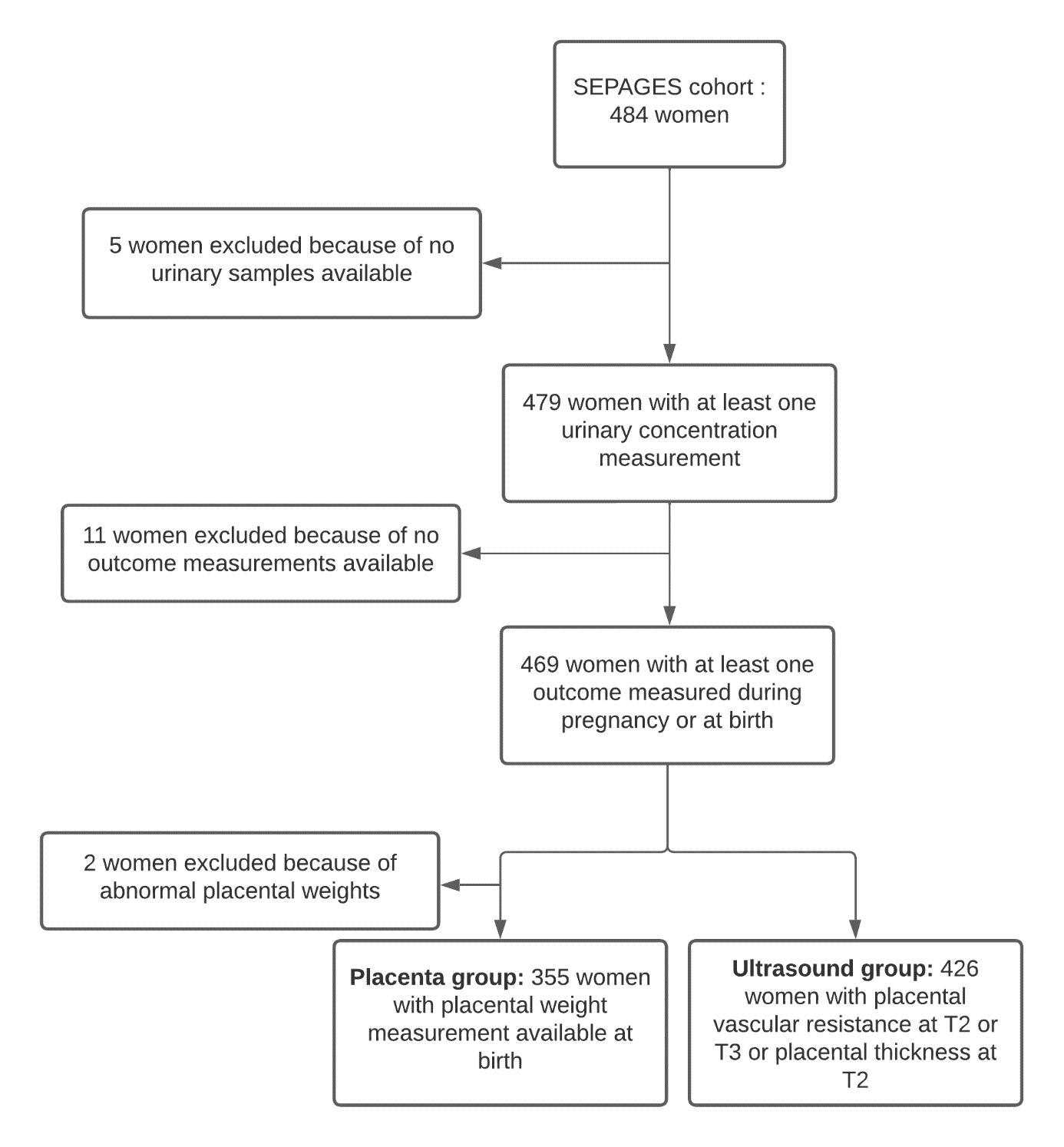


Supplementary Figure S1 – Flowchart of the study population selection


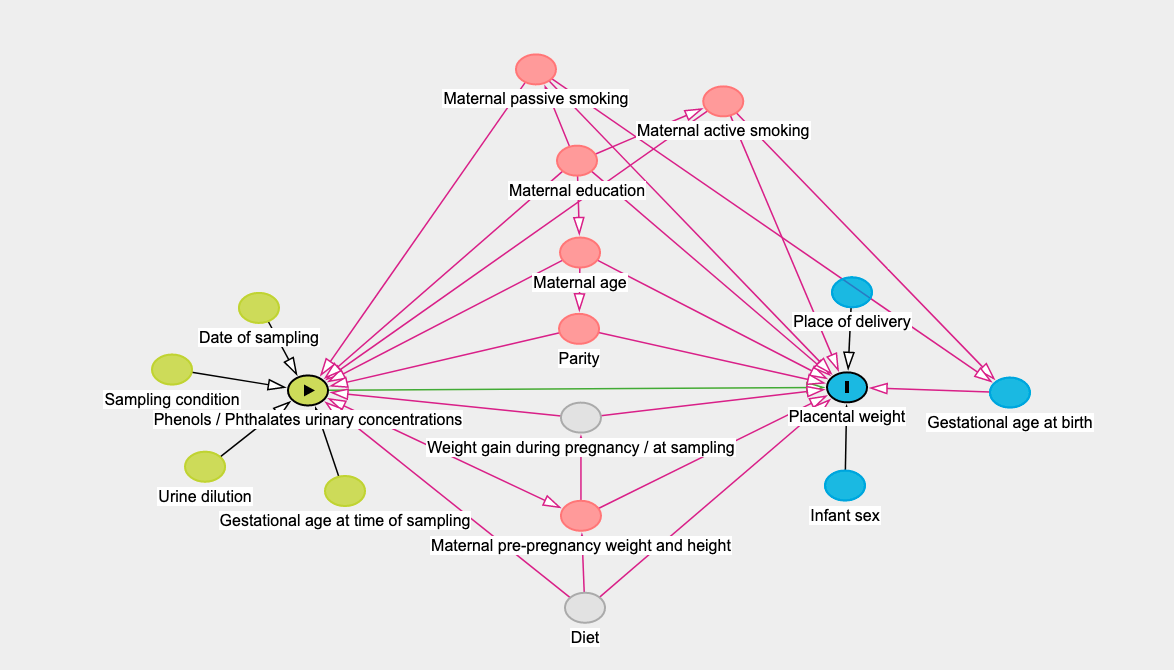


Supplementary Figure S2 - Directional Acyclic Graph for placental weight

*Made with DAGitty (*[*http://www.dagitty.net/*](http://www.dagitty.net/)*)*

*Green: Exposure predictors; Blue: Outcome predictors; Pink: Confounders; Grey: Unobserved confounders.*


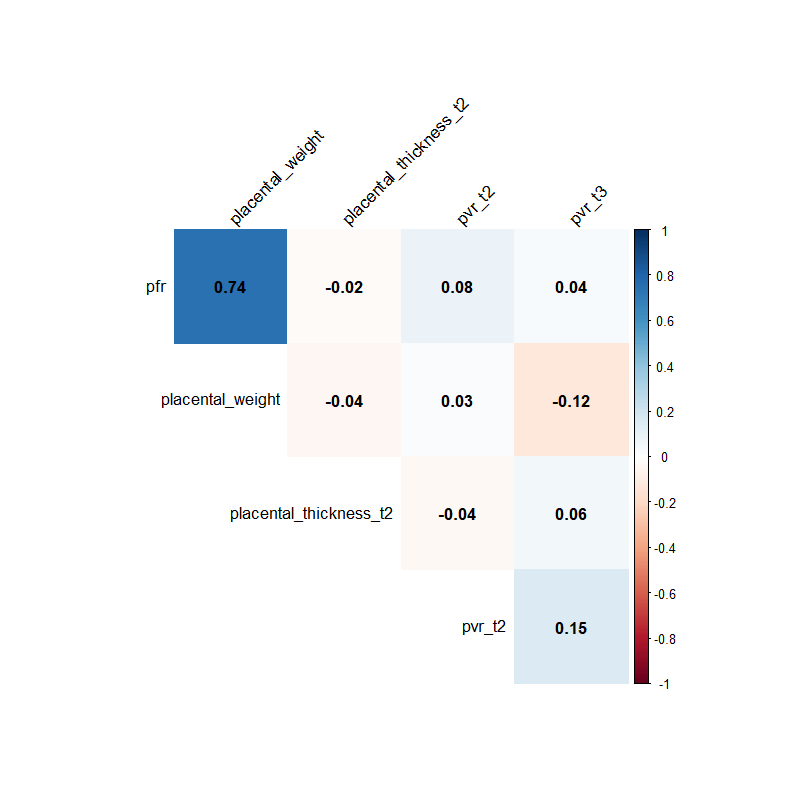


Supplementary Figure S3 – Outcomes correlation plot

The colour gradient on the right represents the range of the correlation’s rho. N = 355.

Abbreviation: PFR: Placental-to-foetal ratio; PVR: Placental vascular resistance; T2: 2^nd^ trimester; T3: 3^rd^ trimester


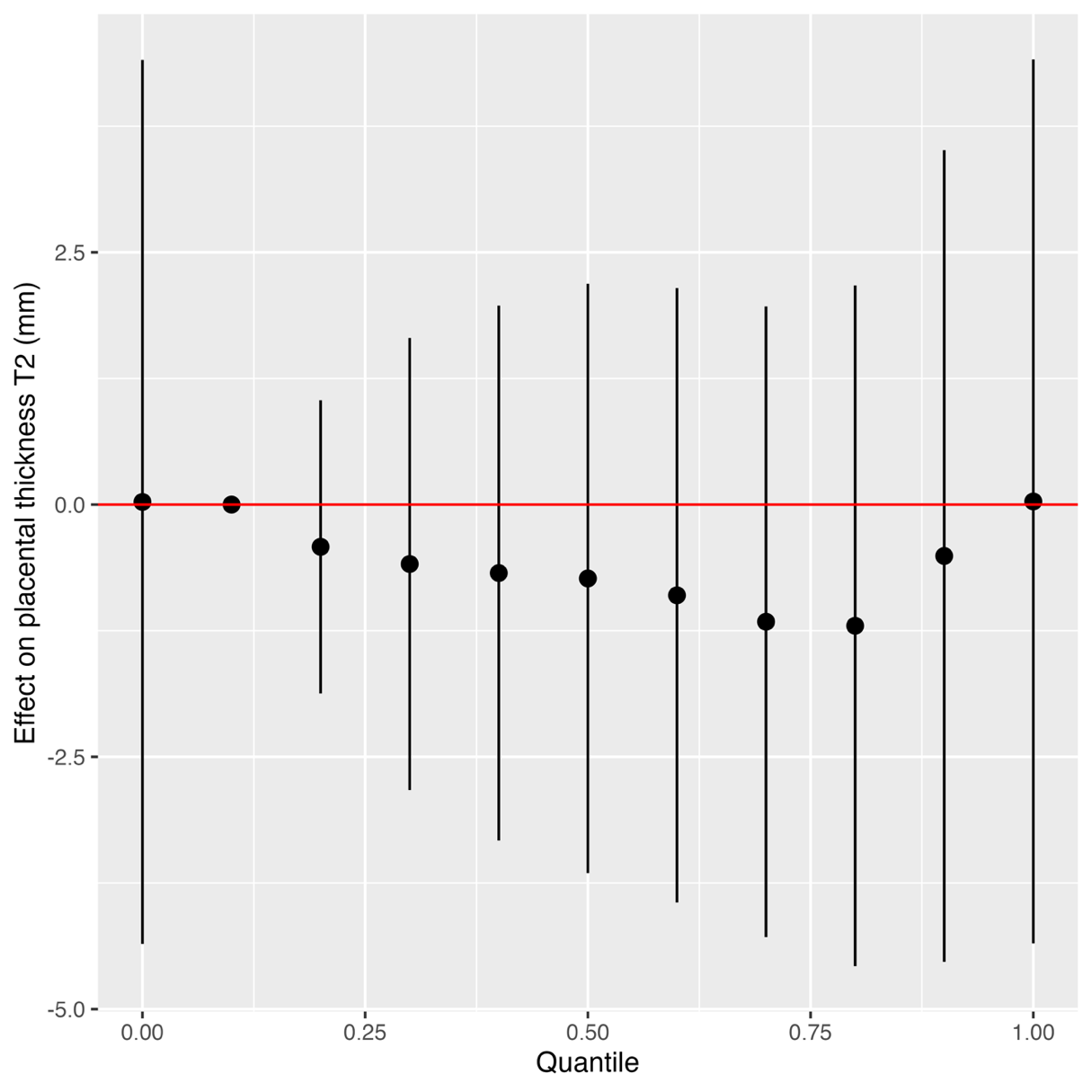


**Supplementary Figure S4 - BKMR Analysis: Quantile-based mixture effect on placental thickness at the second trimester.** Each dot represents the expected change in outcome when all the compounds of the mixture are at the quantiles displayed on the X axis. The range of quantiles was from the 0^th^ to the 100^th^, with the reference being the 10^th^ percentile and each dots representing an increase by 10 percentiles. The redline represents the null, and the black vertical lines are the 95% confidence intervals.


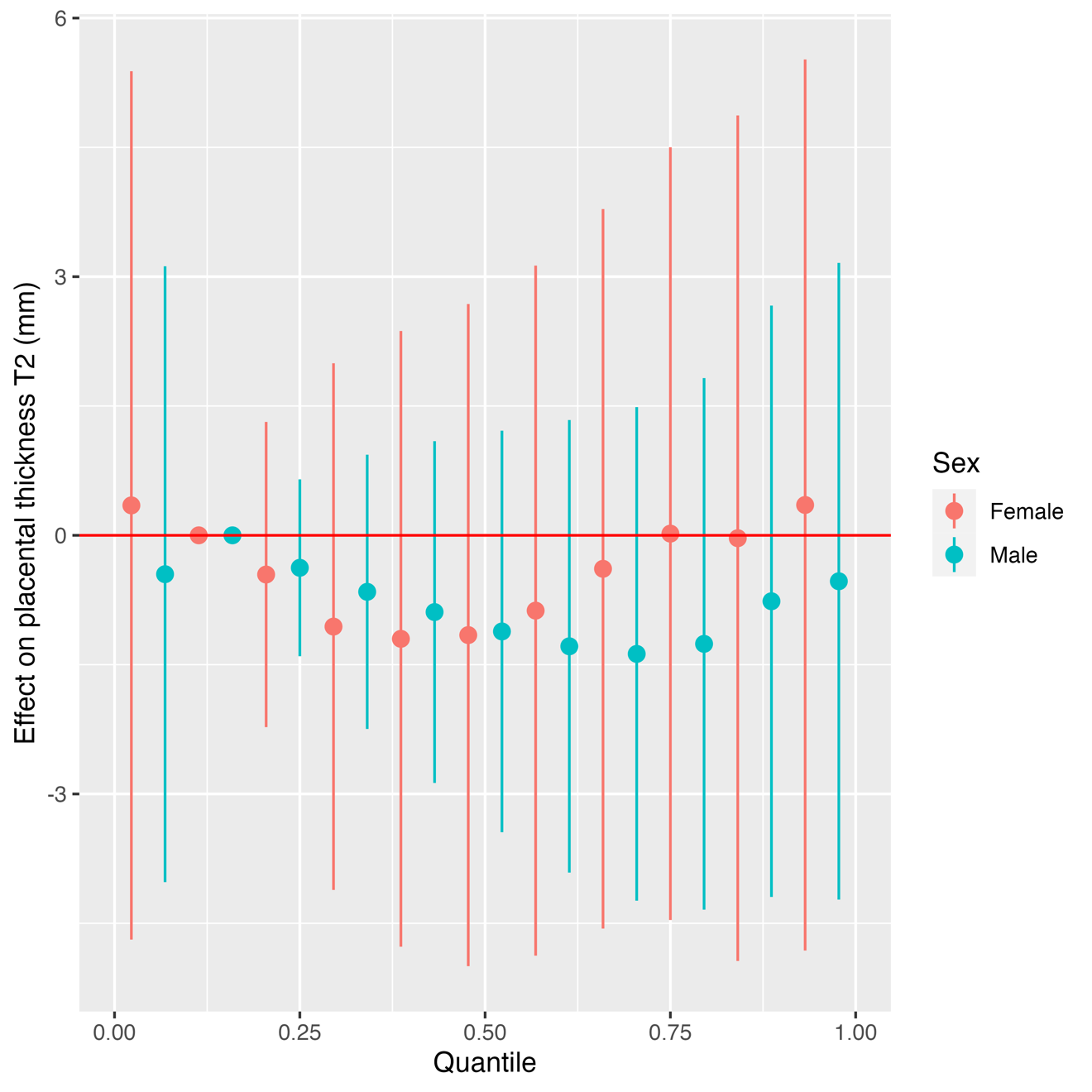


**Supplementary Figure S5 - BKMR Analysis: Quantile-based mixture effect on placental thickness at the second trimester with sex-stratified population.** Each dot represents the expected change in outcome when all the compounds of the mixture are at the quantiles displayed on the X axis. The range of quantiles was from the 0^th^ to the 100^th^, with the reference being the 10^th^ percentile and each dots representing an increase by 10 percentiles. The redline represents the null, and the black vertical lines are the 95% confidence intervals.


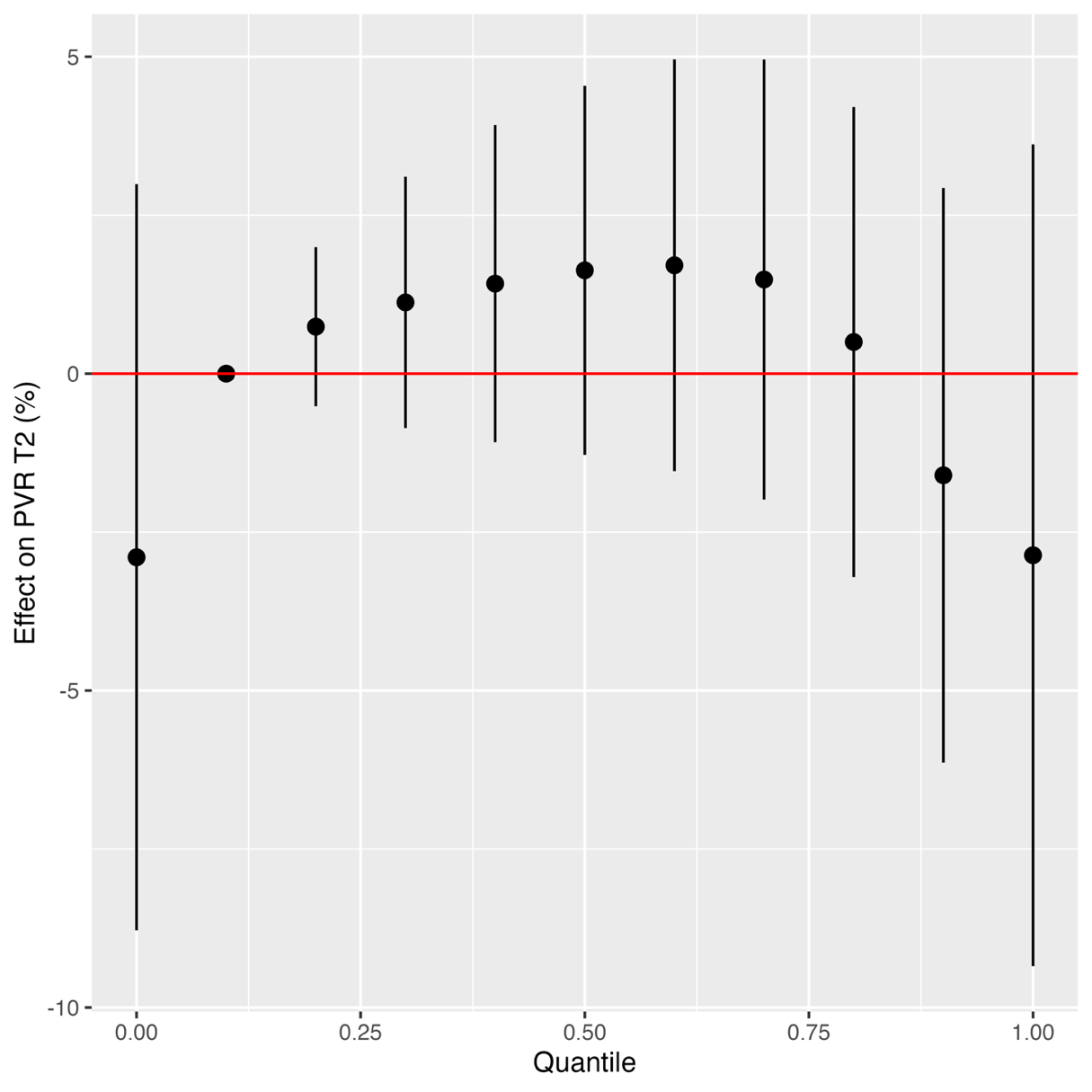


**Supplementary Figure S6 - BKMR Analysis: Quantile-based mixture effect on placental vascular resistance at the second trimester.** Each dot represents the expected change in outcome when all the compounds of the mixture are at the quantiles displayed on the X axis. The range of quantiles was from the 0^th^ to the 100^th^, with the reference being the 10^th^ percentile and each dots representing an increase by 10 percentiles. The redline represents the null, and the black vertical lines are the 95% confidence intervals.


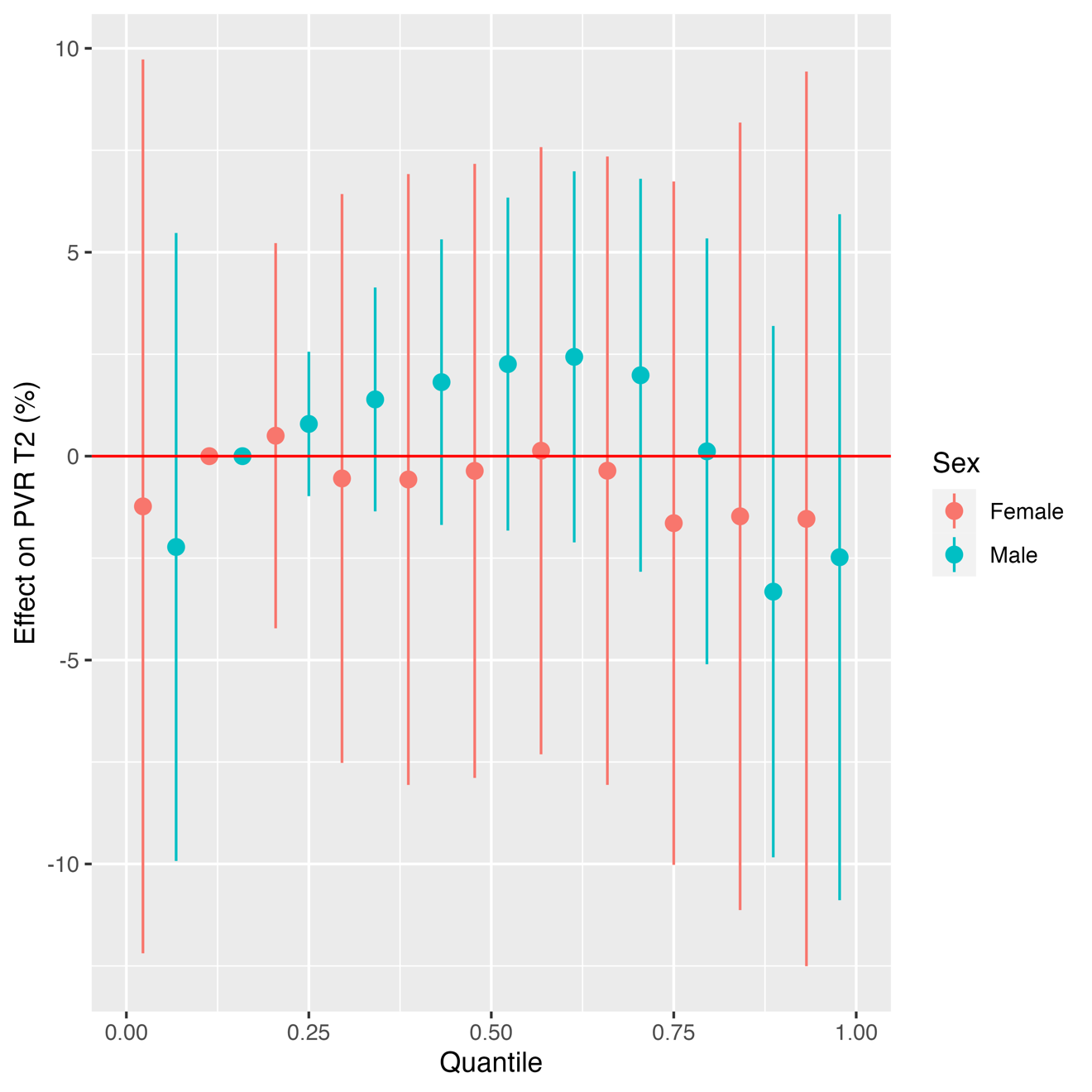


**Supplementary Figure S7 - BKMR Analysis: Quantile-based mixture effect on placental vascular resistance at the second trimester with sex-stratified population.** Each dot represents the expected change in outcome when all the compounds of the mixture are at the quantiles displayed on the X axis. The range of quantiles was from the 0^th^ to the 100^th^, with the reference being the 10^th^ percentile and each dots representing an increase by 10 percentiles. The redline represents the null, and the black vertical lines are the 95% confidence intervals.


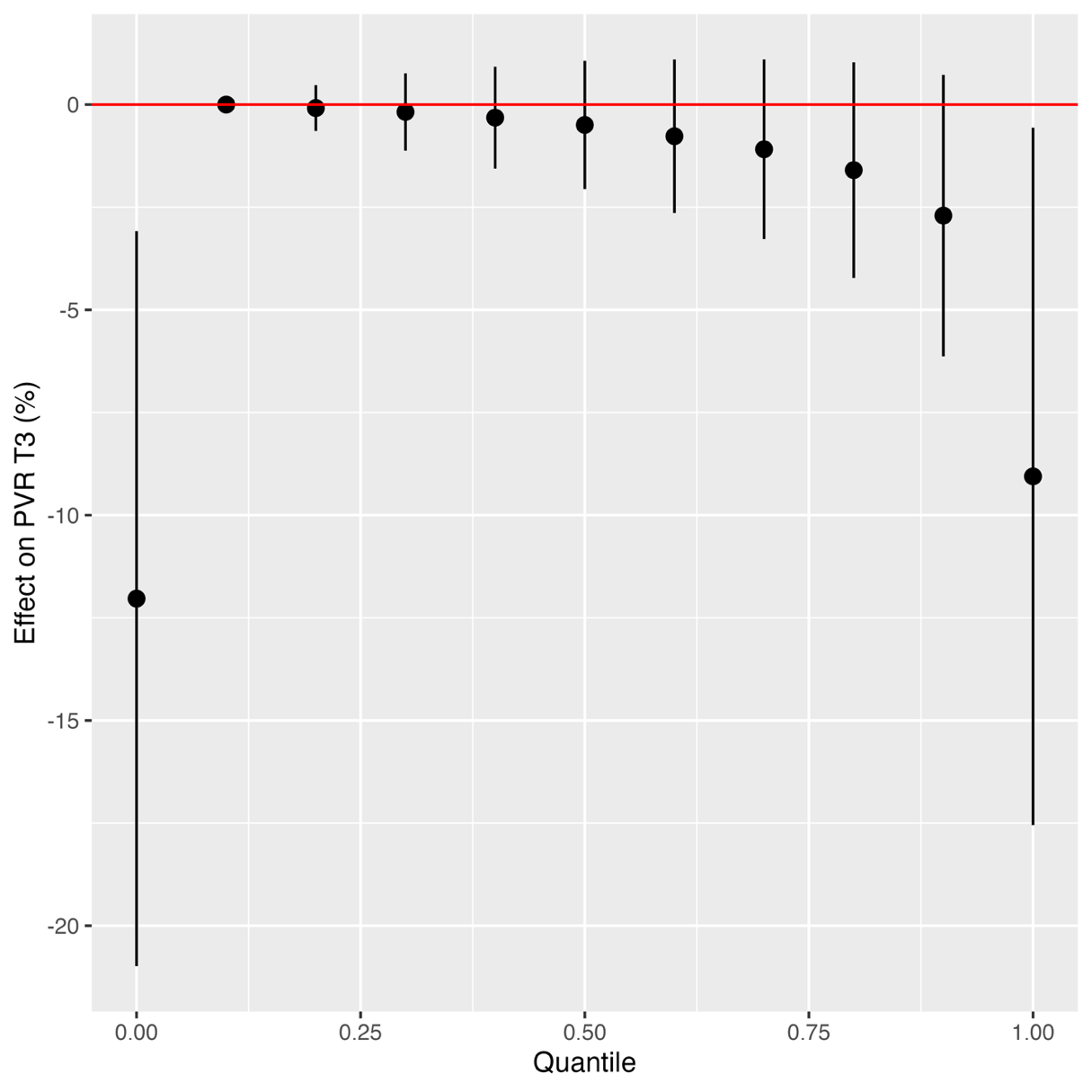


**Supplementary Figure S8 - BKMR Analysis: Quantile-based mixture effect on placental vascular resistance at the third trimester.** Each dot represents the expected change in outcome when all the compounds of the mixture are at the quantiles displayed on the X axis. The range of quantiles was from the 0^th^ to the 100^th^, with the reference being the 10^th^ percentile and each dots representing an increase by 10 percentiles. The redline represents the null, and the black vertical lines are the 95% confidence intervals.


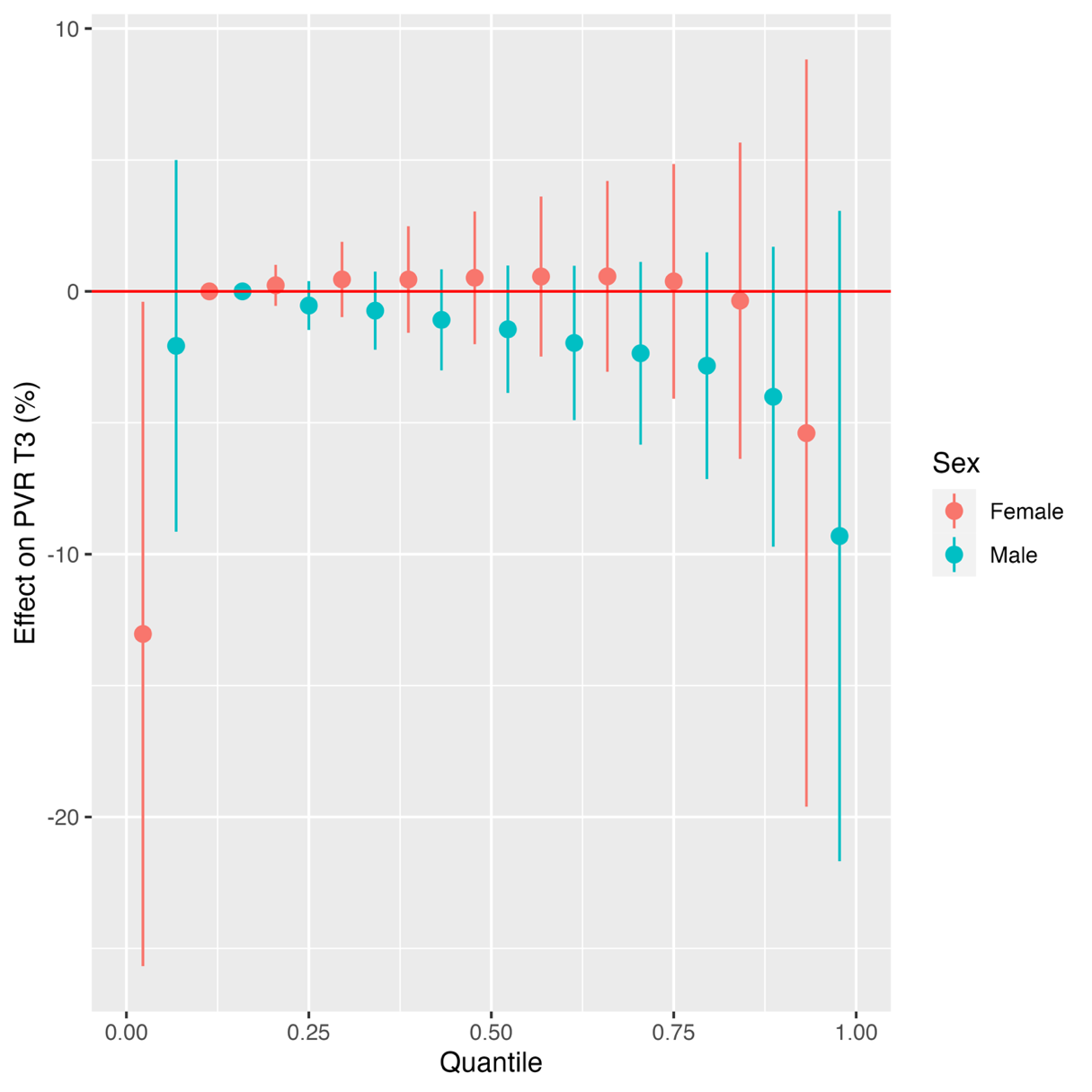


**Supplementary Figure S9 - BKMR Analysis: Quantile-based mixture effect on placental vascular resistance at the third trimester with sex-stratified population.** Each dot represents the expected change in outcome when all the compounds of the mixture are at the quantiles displayed on the X axis. The range of quantiles was from the 0^th^ to the 100^th^, with the reference being the 10^th^ percentile and each dots representing an increase by 10 percentiles. The redline represents the null, and the black vertical lines are the 95% confidence intervals


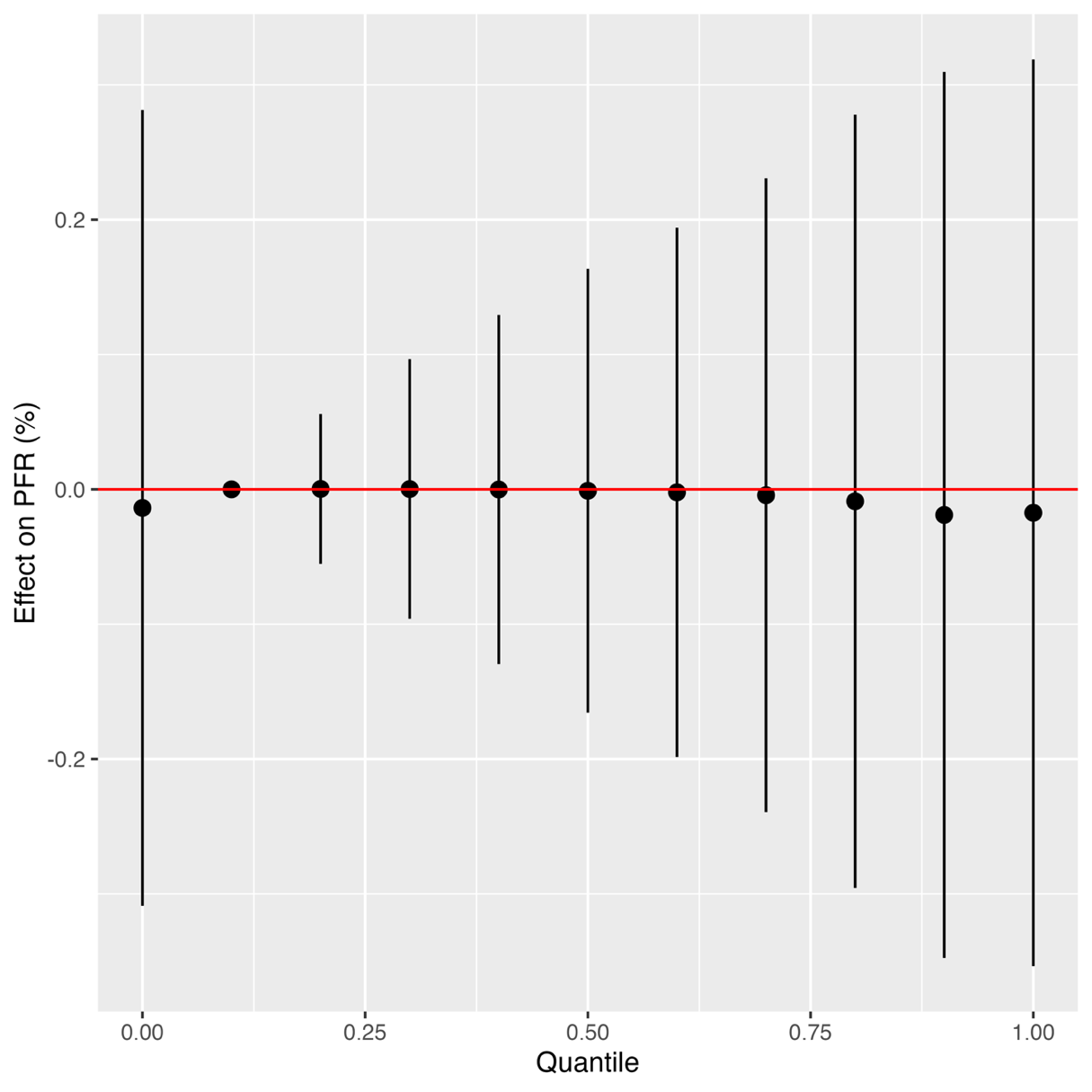


**Supplementary Figure S10 - BKMR Analysis: Quantile-based mixture effect on PFR.** Each dot represents the expected change in outcome when all the compounds of the mixture are at the quantiles displayed on the X axis. The range of quantiles was from the 0^th^ to the 100^th^, with the reference being the 10^th^ percentile and each dots representing an increase by 10 percentiles. The redline represents the null, and the black vertical lines are the 95% confidence intervals.


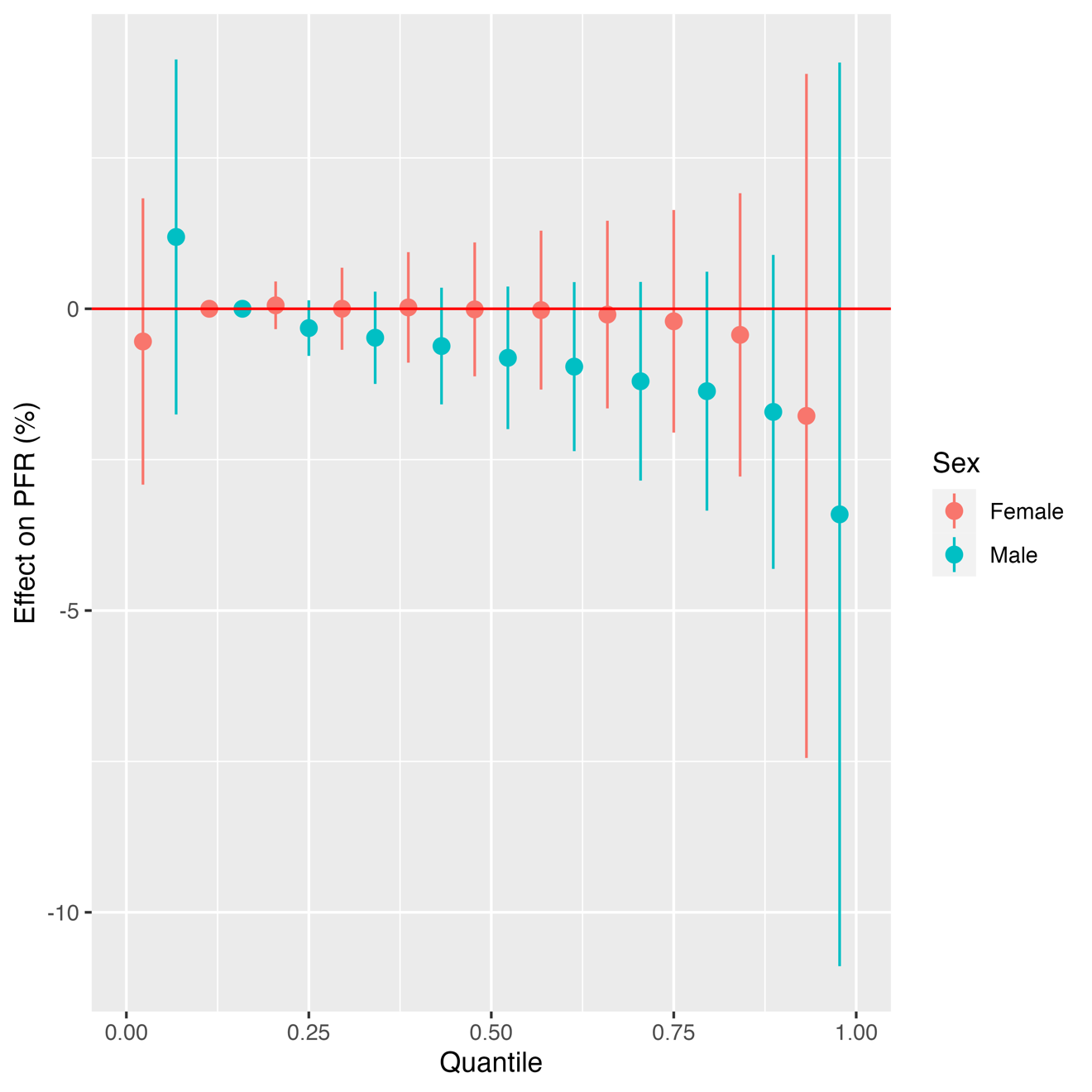


**Supplementary Figure S11 - BKMR Analysis: Quantile-based mixture effect on PFR with sex-stratified population.** Each dot represents the expected change in outcome when all the compounds of the mixture are at the quantiles displayed on the X axis. The range of quantiles was from the 0^th^ to the 100^th^, with the reference being the 10^th^ percentile and each dots representing an increase by 10 percentiles. The redline represents the null, and the black vertical lines are the 95% confidence intervals


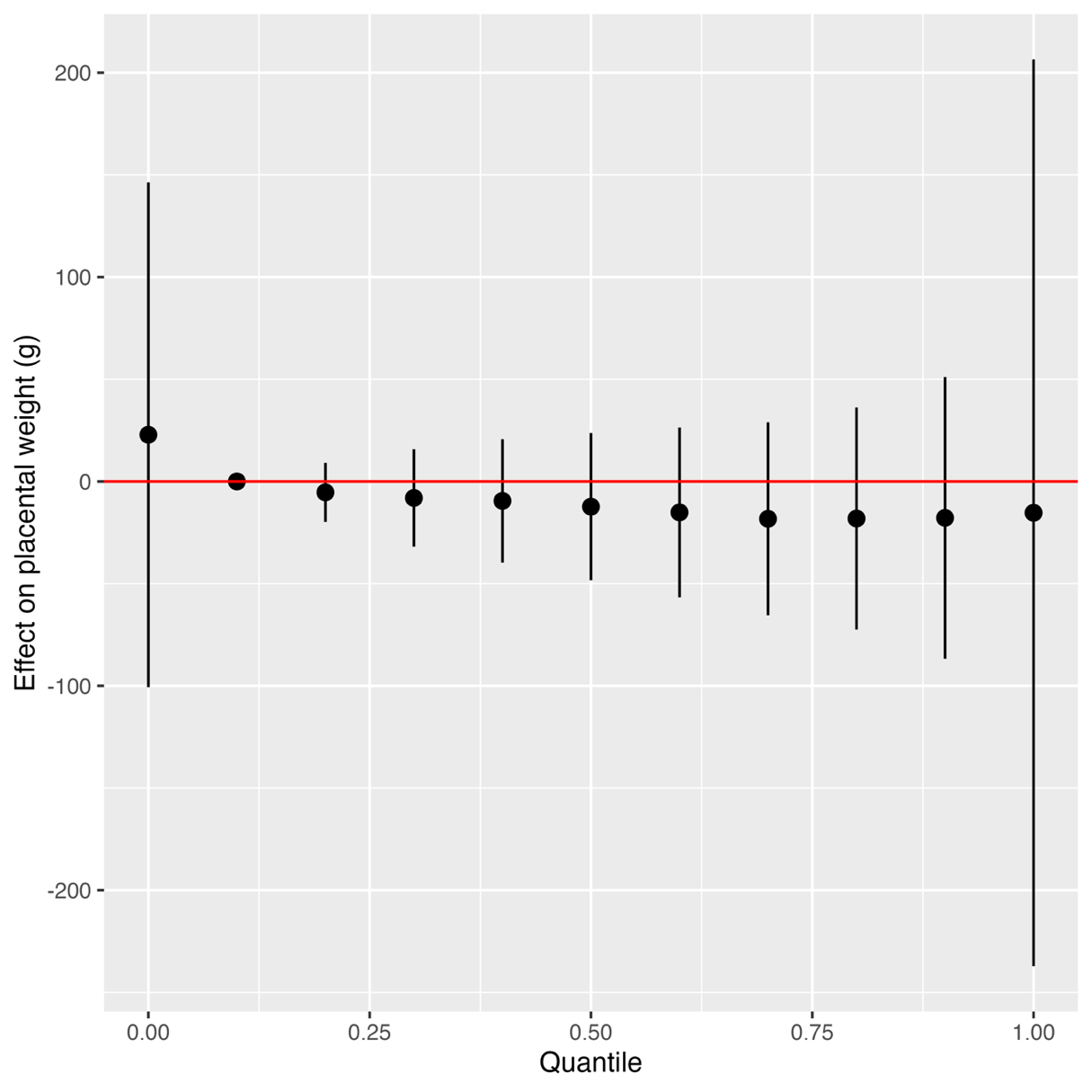


**Supplementary Figure S12 - BKMR Analysis: Quantile-based mixture effect on placental weight.** Each dot represents the expected change in outcome when all the compounds of the mixture are at the quantiles displayed on the X axis. The range of quantiles was from the 0^th^ to the 100^th^, with the reference being the 10^th^ percentile and each dots representing an increase by 10 percentiles. The redline represents the null, and the black vertical lines are the 95% confidence intervals.


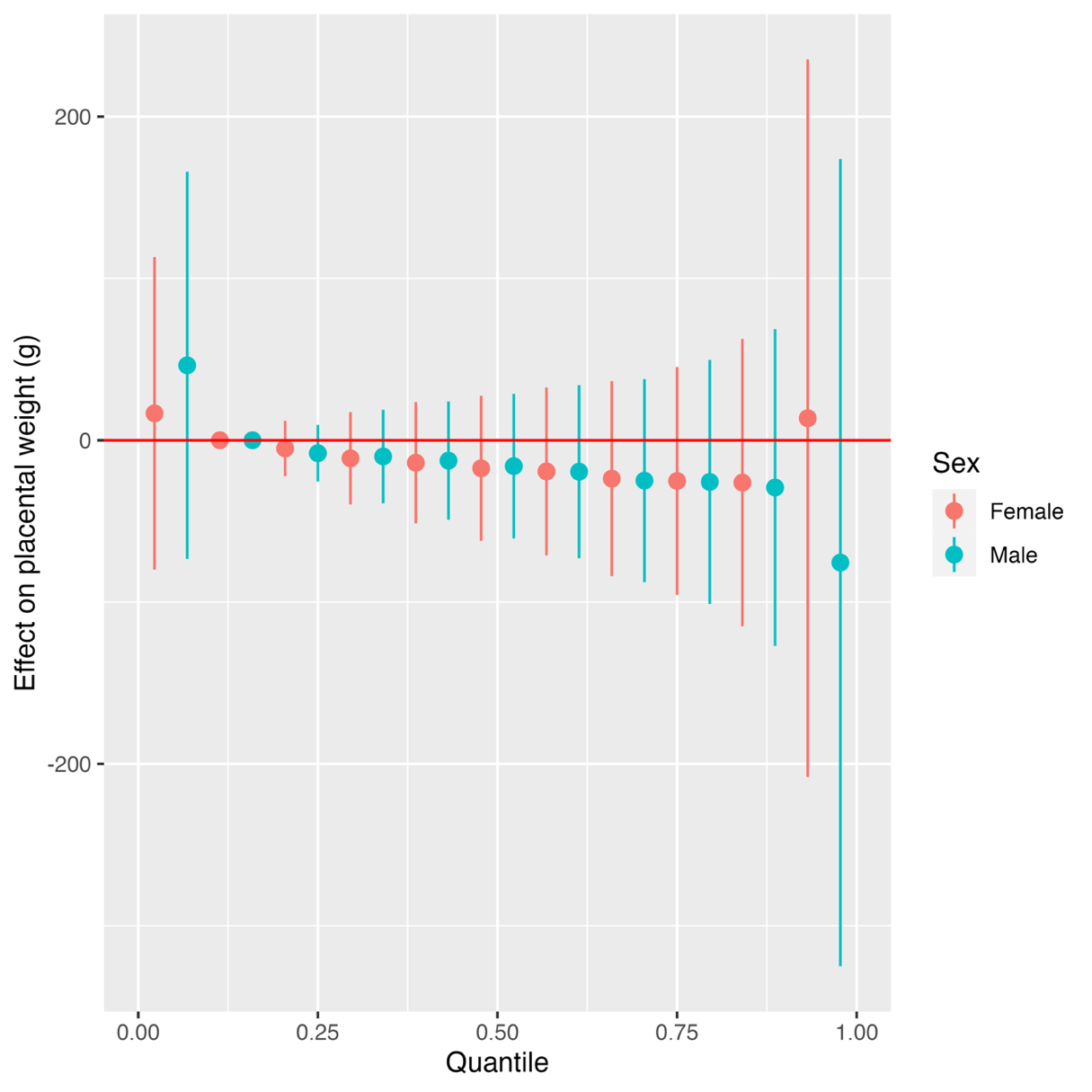


**Supplementary Figure S13 - BKMR Analysis: Quantile-based mixture effect on placental weight with sex-stratified population.** Each dot represents the expected change in outcome when all the compounds of the mixture are at the quantiles displayed on the X axis. The range of quantiles was from the 0^th^ to the 100^th^, with the reference being the 10^th^ percentile and each dots representing an increase by 10 percentiles. The redline represents the null, and the black vertical lines are the 95% confidence intervals.

Supplementary tables

Supplementary Table S1 – Standardization parameters for each exposure variable

| Compound | Analytical batch | Transport duration | Defrosting duration during the pooling procedure |
| --- | --- | --- | --- |
| Bisphenol A | X | X |  |
| Methyl paraben | X |  | X |
| Ethyl paraben | X |  | X |
| Propyl paraben | X |  | X |
| Triclosan |  |  |  |
| Benzophenone-3 | X | X |  |
| MEP | X |  |  |
| MnBP | X |  |  |
| MBzP |  | X |  |
| MiBP | X | X |  |
| OH-MPHP | X |  |  |
| MEOHP | X |  |  |
| MECPP | X |  |  |
| MEHHP | X |  |  |
| MEHP | X |  |  |
| MMCHP | X |  |  |
| cx-MiNP | X |  |  |
| oh-MiNP |  |  |  |
| oh-MINCH | X |  |  |
| oxo-MINCH | X |  | X |
| oxo-MiNP | X |  |  |

Abbreviations: MEP: Monoethyl phthalate; MnBP: Mono-n-butyl phthatale; MBzP : Monobenzyl phthalate; MiBP: Mono-isobutyl phthalate; OH-MPHP: 6-hydroxy-mono-propyl-heptyl phthalate; MEOHP: Mono(2-ethyl-5oxohexyl) phthalate; MECPP: Mono(2-ethyl-5-carboxypentyl) phthalate; MEHHP: Mono(2-ethyl-5-hydroxyhexyl) phthalate; MEHP: Mono(2-ethylhexyl) phthalate; MMCHP: Mono-2-carboxymethyl hexyl phthalate; cx-MiNP: Mono-4-methyl-7-carboxyoctyl phthalate; oh-MiNP: Mono-4-methyl-7-hydroxyoctyl phthalate; oxo-MiNP: Mono-4-methyl-7-oxooctyl phthalate; oh-MINCH: 2-(((Hydroxy-4-methyloctyl)oxy)carbonyl)cyclohexanecarboxylic Acid; oxo-MINCH: 2-(((4-Methyl-7-oxyooctyl)oxy)carbonyl)cyclohexanecarboxylic Acid

Supplementary Table S2 – Molecular weights used in molar sum computation

| **Parent compound** | **Metabolite** | **Molecular weight (g/mol)** |
| --- | --- | --- |
| Di-isononyl phthalate (DiNP) | oh-MiNP | 308.2 |
|  | oxo-MiNP | 306.2 |
|  | cx-MiNP | 322.2 |
| 1,2-Cyclohexane dicarboxylic acid diisononyl ester (DINCH) | oh-MINCH | 314.2 |
|  | oxo-MINCH | 312.4 |
| Bis(2-ethylhexyl) phthalate (DEHP) | MEOHP | 292 |
|  | MECPP | 308 |
|  | MEHP | 278 |
|  | MEHHP | 294 |
|  | MCMHP | 308.3 |

Abbreviation: oh-MiNP: Mono-hydroxy-isononyl phthalate; oxo-MiNP: Mono-oxo-isononyl phthalate; cx-MiNP: Mono-carboxy-isononyl phthalate; MEOHP: Mono(2-ethyl-5-oxohexyl) phthalate; MECPP: Mono(2-ethyl-5-carboxypentyl) phthalate; MEHP: Mono(2-ethylhexyl) phthalate; MEHHP: Mono(2-ethyl-5-hydroxyhexyl) phthalate; MCMHP: Mono(2-carboxymethylhexyl) phthalate

Supplementary Table S3 – Population characteristics between women with and without placental weight measurement available at birth

| **Characteristic** | **No placental weight measurement**, N = 127  Median (IQR); n (%) | **Placental weight measurement**, N = 357  Median (IQR); n (%) | **p-value^1^** |
| --- | --- | --- | --- |
| **Age at conceptions, years** | 31.9 (30.0, 35.5) | 32.2 (29.9, 35.0) | 0.9 |
| **Maternal BMI before pregnancy (kg/m2)** | 21.8 (19.8, 24.0) | 21.3 (19.7, 23.9) | 0.3 |
| Unknown | 1 | 3 |  |
| **Gestational duration, LMP-based, weeks** | 40.00 (38.50, 40.71) | 40.00 (39.14, 40.86) | 0.14 |
| Unknown | 4 | 0 |  |
| **Parity** |  |  | 0.7 |
| Nulliparous | 60 (47%) | 162 (45%) |  |
| Uni/Multiparous | 67 (53%) | 195 (55%) |  |
| **Maternity ward** |  |  | <0.001 |
| 1 | 55 (45%) | 106 (30%) |  |
| 2 | 37 (31%) | 136 (38%) |  |
| 3 | 18 (15%) | 93 (26%) |  |
| 4 | 4 (3.3%) | 22 (6.2%) |  |
| 5 | 7 (5.8%) | 0 (0%) |  |
| Unknown | 6 | 0 |  |
| **Education level** |  |  | 0.9 |
| Up to two years | 20 (16%) | 63 (18%) |  |
| Between 3 and 4 years | 35 (28%) | 92 (26%) |  |
| Five years or more | 72 (57%) | 200 (56%) |  |
| Unknown | 0 | 2 |  |
| **Exposed to passive smoking during pregnancy** |  |  | 0.3 |
| No | 81 (76%) | 271 (81%) |  |
| Yes | 25 (24%) | 63 (19%) |  |
| Unknown | 21 | 23 |  |
| **Smoked during pregnancy** |  |  | 0.4 |
| No | 100 (95%) | 301 (93%) |  |
| Yes | 5 (4.8%) | 24 (7.4%) |  |
| Unknown | 22 | 32 |  |
| **Mode of delivery** |  |  | <0.001 |
| Non-instrumental VD | 80 (66%) | 260 (73%) |  |
| Instrumental VD | 8 (6.6%) | 50 (14%) |  |
| Planned C-section | 7 (5.7%) | 19 (5.3%) |  |
| Emergency C-section | 27 (22%) | 28 (7.8%) |  |
| Unknown | 5 | 0 |  |
| Median (IQR); n (%)  ^1^Wilcoxon rank sum test; Pearson's Chi-squared test; Fisher's exact test  VD: Vaginal delivery | | |  |

Supplementary Table S4 – Distribution of the placental parameter assessed during pregnancy and at birth

|  |  |  | Percentiles | | | | |
| --- | --- | --- | --- | --- | --- | --- | --- |
| Outcome | N | Mean±SD | 5th | 25th | 50th | 75th | 95th |
| Placental thickness T2 (mm) | 392 | 25.3±5.95 | 17 | 21.42 | 24.55 | 28 | 36.55 |
| PVR T2 (%) | 380 | 71.3±6.88 | 60 | 67 | 72 | 76 | 81 |
| PVR T3 (%) | 388 | 63.76±7.44 | 50 | 59 | 64 | 69 | 75 |
| Placental weight (g) | 355 | 535.58±112.29 | 367 | 460 | 530 | 600 | 720 |
| PFR (%) | 355 | 16.22±2.93 | 11.87 | 14.45 | 16.1 | 17.7 | 20.83 |

Abbreviations: PFR: Placental to foetal ratio, PVR: Placental vascular resistance

Supplementary Table S5 – Adjusted associations between urinary biomarkers in terciles and placental weight

|  |  | **Urinary concentration at 2^nd^ Trimester** | | | |  | **Urinary concentration at 3^rd^ Trimester** | | | |
| --- | --- | --- | --- | --- | --- | --- | --- | --- | --- | --- |
| **Chemical** | **Tercile** | **Estimate** | **95%CI** | **Hetero pv** | **Trend pv** |  | **Estimate** | **95%CI** | **Hetero pv** | **Trend pv** |
| Bisphenol A | 2nd | -25,1 | (-54.8;4.6) | 0.23 | 0.77 |  | -10,7 | (-40.9;19.5) | 0.59 | 0.33 |
|  | 3rd | -6 | (-35.2;23.3) |  |  |  | -15,5 | (-45.8;14.9) |  |  |
| Methyl paraben | 2nd | -7,3 | (-36.8;22.2) | 0.19 | 0.16 |  | 23,7 | (-6.1;53.5) | 0.15 | 0.11 |
|  | 3rd | 19,2 | (-10.6;48.9) |  |  |  | 27,2 | (-2.9;57.2) |  |  |
| Ethyl paraben | 2nd | 2,4 | (-27.1;31.8) | 0.97 | 0.88 |  | -20,6 | (-50.3;9) | 0.15 | 0.38 |
|  | 3rd | -1,6 | (-31.9;28.7) |  |  |  | 8 | (-22;38) |  |  |
| Propyl paraben | 2nd | -17,73 | (-47.6;12.2) | 0.5 | 0.77 |  | -14,6 | (-44.9;15.6) | 0.61 | 0.49 |
|  | 3rd | -5,9 | (-35.5;23.8) |  |  |  | -11,4 | (-41.6;18.9) |  |  |
| Benzophenone-3 | 2nd | 7,8 | (-21.8;37.3) | 0.8 | 0.55 |  | 21 | (-8.7;50.6) | 0.36 | 0.4 |
|  | 3rd | 9,6 | (-21;40.2) |  |  |  | 15,7 | (-15;46.4) |  |  |
| Triclosan | 2nd | 2,2 | (-27.6;31.9) | 0.32 | 0.17 |  | -3,2 | (-33.2;26.9) | 0.11 | 0.06 |
|  | 3rd | -18,5 | (-48.5;11.5) |  |  |  | 26,1 | (-4.1;56.2) |  |  |
| MEP | 2nd | 2,7 | (-27.3;32.7) | 0.98 | 0.94 |  | -7,3 | (-37.2;22.5) | 0.76 | 0.47 |
|  | 3rd | 1,1 | (-29.9;32) |  |  |  | -11,4 | (-42.6;19.8) |  |  |
| MnBP | 2nd | -15,7 | (-45.1;13.6) | 0.19 | 0.08 |  | -7 | (-36.8;22.8) | 0.89 | 0.92 |
|  | 3rd | -27,6 | (-57.7;2.6) |  |  |  | -1,6 | (-32.4;29.3) |  |  |
| MiBP | 2nd | -9,9 | (-39.3;19.4) | 0.68 | 0.42 |  | -6,8 | (-36.4;22.9) | 0.12 | 0.13 |
|  | 3rd | -12,9 | (-43.5;17.7) |  |  |  | 22,9 | (-7.6;53.4) |  |  |
| MBzP | 2nd | -34,8 | (-64.3; -5.2) | 0.04 | 0.04 |  | -11,3 | (-41;18.4) | 0.45 | 0.21 |
|  | 3rd | -32,1 | (-62.3; -1.9) |  |  |  | -19,3 | (-49.5;10.8) |  |  |
| OH-MPHP | 2nd | -22,6 | (-52.2;7) | 0.23 | 0.84 |  | -11,4 | (-41.7;18.9) | 0.76 | 0.68 |
|  | 3rd | -1,4 | (-32;29.2) |  |  |  | -7 | (-37.4;23.4) |  |  |
| ∑DEHP | 2nd | -18,4 | (-47.7;10.9) | 0.29 | 0.16 |  | -12,7 | (-42.5;17) | 0.43 | 0.65 |
|  | 3rd | -22,4 | (-52.6;7.7) |  |  |  | 6,8 | (-23.1;36.7) |  |  |
| ∑DiNP | 2nd | 11,7 | (-18;41.4) | 0.59 | 0.35 |  | -15,3 | (-45.7;15.1) | 0.57 | 0.99 |
|  | 3rd | 15,1 | (-15.1;45.3) |  |  |  | -2,6 | (-32.9;27.7) |  |  |
| ∑DINCH | 2nd | 38,3 | (8.3;68.2) | 0.04 | 0.15 |  | -3,4 | (-33.2;26.4) | 0.97 | 0.87 |
|  | 3rd | 27,6 | (-2.2;57.4) |  |  |  | -2,7 | (-32.9;27.4s) |  |  |

Abbreviation: Hetero pv: Heterogeneity p-value (Wald test); pv: p-value; ∑: Molar sum; MEP: Monoethyl phthalate; MnBP: Mono-n-butyl phthalate; MiBP: Mono-isobutyl phthalate; MBzP: Monobenzyl phthalate; OH-MPHP: 6-hydroxy-mono-propyl-heptyl phthalate; DINCH: Di(isononyl)cyclohexane-1,2-dicarboxylate; DiNP: Diisononyl phthalate; DEHP: Di(2-ethylhexyl) phthalate

Adjustment factors: Maternal age, parity, education level, active smoking, passive smoking, BMI before pregnancy, gestational duration, maternity ward

Supplementary Table S6 – Adjusted associations between standardized phenols and phthalates urinary concentrations and placental outcomes in the whole population.

|  |  | **Placental thickness T2** | | **PVR T2** | | **PVR T3** | | **PFR** | | **Placental weight** | |
| --- | --- | --- | --- | --- | --- | --- | --- | --- | --- | --- | --- |
| **Chemical** | **Trimester** | **β (95%CI)** | **Inter. pv** | **β (95%CI)** | **Inter. pv** | **β (95%CI)** | **Inter. pv** | **β (95%CI)** | **Inter. pv** | **β (95%CI)** | **Inter. pv** |
| Bisphenol A | T2 | -0.61 (-1.3; 0.08) | 0,9 | 0.39 (-0.39; 1.17) | 0,7 | -0.17 (-1.04; 0.69) | 0,71 | -0.12 (-0.48; 0.24) | 0,68 | -9 (-23; 5) | 0,44 |
|  | T3 |  |  |  |  | 0.28 (-0.48; 1.04) | 0,87 | -0.16 (-0.48; 0.16) | 0,29 | -7.93 (-20.16; 4.31) | 0,54 |
| Bisphenol S LOD-LOQ | T2 | -0.32 (-3.42; 2.78) | 0,63 | 0.03 (-3.63; 3.69) | 0,93 | 1.12 (-2.6; 4.85) | 0,43 | -1.43 (-3.04; 0.18) | 0,91 | -57.58 (-119.85; 4.68) | 0,71 |
|  | T3 |  |  |  |  | 0.45 (-2.91; 3.8) | 0,84 | -0.61 (-1.87; 0.65) | 0,51 | -27.02 (-75.34; 21.3) | 0,81 |
| Bisphenol S >LOQ | T2 | 1.28 (-0.19; 2.74) | 0,15 | 0.54 (-1.1; 2.18) | 0,72 | -0.14 (-1.97; 1.68) | 0,64 | -0.64 (-1.4; 0.13) | 0,37 | 3.4 (-26.17; 32.96) | 0,26 |
|  | T3 |  |  |  |  | -0.21 (-2.05; 1.64) | 0,82 | 0.08 (-0.69; 0.85) | 0,57 | 5.85 (-23.7; 35.41) | 0,51 |
| Methyl paraben | T2 | -0.01 (-0.43; 0.4) | 0,97 | 0.21 (-0.25; 0.68) | 0,27 | 0.3 (-0.22; 0.82) | 0,3 | -0.02 (-0.23; 0.2) | 0,61 | 1.75 (-6.35; 9.85) | 0,4 |
|  | T3 |  |  |  |  | -0.04 (-0.43; 0.34) | 0,27 | 0.04 (-0.12; 0.19) | 0,69 | 2.12 (-3.97; 8.2) | 0,55 |
| Ethyl paraben | T2 | 0.23 (-0.18; 0.63) | 0,98 | -0.2 (-0.64; 0.24) | 0,56 | 0.09 (-0.42; 0.59) | 0,77 | -0.04 (-0.24; 0.17) | 0,82 | -0.63 (-8.51; 7.25) | 0,46 |
|  | T3 |  |  |  |  | 0.39 (-0.12; 0.9) | 0,32 | 0.08 (-0.13; 0.28) | 0,19 | 3.51 (-4.5; 11.53) | 0,46 |
| Propyl paraben | T2 | -0.07 (-0.28; 0.15) | 0,86 | 0.06 (-0.18; 0.31) | 0,02 | -0.12 (-0.38; 0.15) | 0,87 | -0.01 (-0.12; 0.09) | 0,68 | -1.09 (-5.21; 3.04) | 0,73 |
|  | T3 |  |  |  |  | -0.2 (-0.43; 0.04) | 0,62 | -0.01 (-0.11; 0.09) | 0,71 | -1.25 (-5.05; 2.54) | 0,91 |
| Butyl paraben LOD-LOQ | T2 | -0.01 (-1.87; 1.86) | 0,1 | -0.91 (-2.94; 1.13) | 0,16 | -0.75 (-3; 1.51) | 0,55 | -0.46 (-1.33; 0.4) | 0,36 | -8.73 (-42.03; 24.57) | 0,39 |
|  | T3 |  |  |  |  | -0.92 (-3.39; 1.55) | 0,45 | -0.31 (-1.29; 0.68) | 0,57 | -24.77 (-62.56; 13.02) | 0,79 |
| Butyl paraben >LOQ | T2 | -0.22 (-2.15; 1.71) | 0,19 | -0.38 (-2.59; 1.82) | 0,23 | -0.88 (-3.31; 1.56) | 0,16 | 0.55 (-0.53; 1.62) | 0,49 | -2.53 (-44.07; 39.02) | 0,5 |
|  | T3 |  |  |  |  | -0.17 (-2.58; 2.24) | 0,78 | 0.74 (-0.26; 1.74) | 0,6 | 3.62 (-34.86; 42.1) | 0,78 |
| Benzophenone-3 | T2 | -0.2 (-0.6; 0.21) | 0,76 | -0.22 (-0.66; 0.21) | 0,23 | 0.22 (-0.27; 0.71) | 0,2 | -0.02 (-0.23; 0.19) | 0,24 | -1.18 (-9.32; 6.95) | 0,17 |
|  | T3 |  |  |  |  | 0.21 (-0.33; 0.75) | 0,5 | 0.07 (-0.17; 0.32) | 0,41 | -0.05 (-9.52; 9.42) | 0,51 |
| Triclosan | T2 | 0.28 (-0.03; 0.59) | 0,49 | -0.09 (-0.42; 0.25) | 0,25 | 0.12 (-0.26; 0.5) | 0,15 | -0.08 (-0.24; 0.08) | 0,91 | -2.98 (-9.04; 3.08) | 0,63 |
|  | T3 |  |  |  |  | 0.15 (-0.24; 0.54) | 0,92 | 0.11 (-0.06; 0.27) | 0,71 | 4.5 (-1.87; 10.87) | 0,93 |
| MEP | T2 | 0.4 (-0.21; 1.01) | 0,42 | -0.2 (-0.89; 0.48) | 0,43 | 0.3 (-0.45; 1.05) | 0,86 | -0.02 (-0.33; 0.29) | 0,26 | -1.07 (-13.07; 10.93) | 0,88 |
|  | T3 |  |  |  |  | 0.23 (-0.52; 0.98) | 0,19 | -0.05 (-0.37; 0.28) | 0,33 | -3.53 (-15.94; 8.88) | 0,33 |
| MnBP | T2 | 0.04 (-0.96; 1.04) | 0,22 | -1.27 (-2.35; -0.18) | 0,45 | -0.87 (-2.12; 0.37) | 0,8 | -0.38 (-0.92; 0.17) | 0,44 | -13.54 (-34.48; 7.41) | 0,68 |
|  | T3 |  |  |  |  | -1.22 (-2.41; -0.03) | 0,6 | -0.12 (-0.6; 0.37) | 0,01 | -1.92 (-20.6; 16.75) | 0,01 |
| MiBP | T2 | -0.6 (-1.58; 0.38) | 0,54 | -0.27 (-1.38; 0.84) | 0,6 | -1.27 (-2.46; -0.07) | 0,31 | -0.04 (-0.56; 0.48) | 0,94 | -4.56 (-24.71; 15.58) | 0,92 |
|  | T3 |  |  |  |  | -1.22 (-2.31; -0.13) | 0,06 | 0.26 (-0.21; 0.72) | 0,4 | 7.92 (-10.02; 25.86) | 0,55 |
| MBzP | T2 | -0.58 (-1.39; 0.23) | 0,81 | -0.47 (-1.39; 0.44) | 0,11 | -0.43 (-1.44; 0.59) | 0,46 | -0.53 (-0.99; -0.07) | 0,82 | -20.13 (-37.76; -2.51) | 0,84 |
|  | T3 |  |  |  |  | -0.86 (-1.84; 0.12) | 0,93 | -0.46 (-0.87; -0.05) | 0,08 | -17.44 (-33.23; -1.64) | 0,05 |
| OH-MPHP | T2 | 0.6 (-0.45; 1.66) | 0,25 | -0.46 (-1.6; 0.68) | 0,32 | -0.14 (-1.48; 1.2) | 0,44 | -0.29 (-0.82; 0.24) | 0,41 | -7.3 (-27.75; 13.15) | 0,77 |
|  | T3 |  |  |  |  | -1.05 (-2.42; 0.31) | 0,33 | 0.17 (-0.36; 0.71) | 0,3 | 6.31 (-14.31; 26.92) | 0,58 |
| ∑DEHP | T2 | -0.56 (-1.55; 0.44) | 0,73 | -0.93 (-2.05; 0.2) | 0,48 | -0.39 (-1.62; 0.84) | 0,89 | -0.18 (-0.7; 0.34) | 0,92 | -8.82 (-28.91; 11.27) | 0,99 |
|  | T3 |  |  |  |  | -0.83 (-2.18; 0.52) | 0,94 | 0.05 (-0.5; 0.6) | 0,37 | 9.01 (-12.26; 30.27) | 0,73 |
| ∑DiNP | T2 | -0.03 (-0.76; 0.69) | 0,8 | 0.2 (-0.6; 1) | 0,72 | -0.56 (-1.45; 0.34) | 0,03 | 0.14 (-0.26; 0.55) | 0,27 | 3.63 (-12.05; 19.3) | 0,93 |
|  | T3 |  |  |  |  | -0.32 (-1.33; 0.7) | 0,62 | -0.22 (-0.64; 0.19) | 0,01 | -2.8 (-18.69; 13.08) | 0,01 |
| ∑DINCH | T2 | -0.04 (-0.69; 0.6) | 0,86 | -0.5 (-1.22; 0.22) | 0,86 | -0.61 (-1.43; 0.21) | 0,21 | 0.32 (-0.02; 0.66) | 0,11 | 11.58 (-1.54; 24.7) | 0,15 |
|  | T3 |  |  |  |  | -0.31 (-1.12; 0.5) | 0,86 | 0.03 (-0.32; 0.37) | 0,82 | 2.41 (-10.84; 15.67) | 0,15 |

Abbreviations: ∑: Molar sum; MEP: Monoethyl phthalate; MnBP: Mono-n-butyl phthalate; MiBP: Mono-isobutyl phthalate; MBzP: Monobenzyl phthalate; OH-MPHP: 6-hydroxy-mono-propyl-heptyl phthalate; DINCH: Di(isononyl)cyclohexane-1,2-dicarboxylate; DiNP: Diisononyl phthalate; DEHP: Di(2-ethylhexyl) phthalate. Inter.pv: Interaction p-value between the exposure and the child’s sex.

Adjustment factors: Maternal age, parity, education level, active smoking, passive smoking, BMI before pregnancy, gestational duration, maternity ward.

Supplementary Table S7 – Adjusted associations between standardized phenols and phthalates urinary concentrations and placental outcomes in sex-stratified population.

|  |  |  | **Placental thickness T2** | **PVR T2** | **PVR T3** | **PFR** | **Placental weight** |
| --- | --- | --- | --- | --- | --- | --- | --- |
| **Chemical** | **Pop.** | **Trimester** | **β (95%CI)** | **β (95%CI)** | **β (95%CI)** | **β (95%CI)** | **β (95%CI)** |
| Propyl paraben | Male | t2 | - | -0.17 (-0.49; 0.15) | - | - | - |
|  | Female | t2 | - | 0.38 (-0.02; 0.77) | - | - | - |
| Butyl paraben LOD-LOQ | Male | t2 | 1.25 (-1.08; 3.59) | - | - | - | - |
|  | Female | t2 | -2.6 (-5.9; 0.7) | - | - | - | - |
| MnBP | Male | t3 | - | - | - | -0.75 (-1.5; -0.01) | -24.28 (-53.31; 4.75) |
|  | Female | t3 | - | - | - | 0.53 (-0.13; 1.19) | 20.82 (-4.36; 45.99) |
| MiBP | Male | t3 | - | - | -0.33 (-1.94; 1.27) | - | - |
|  | Female | t3 | - | - | -1.87 (-3.39; -0.35) | - | - |
| MBzP | Male | t3 | - | - | - | -0.76 (-1.36; -0.15) | -30.86 (-54.28; -7.44) |
|  | Female | t3 | - | - | - | -0.06 (-0.66; 0.54) | 0.12 (-22.81; 23.04) |
| ∑DiNP | Male | t2 | - | - | -1.46 (-2.72; -0.21) | - | - |
|  |  | t3 | - | - | - | -0.81 (-1.44; -0.18) | -24.37 (-49.08; 0.33) |
|  | Female | t2 | - | - | 0.54 (-0.74; 1.82) | - | - |
|  |  | t3 | - | - | - | 0.23 (-0.33; 0.78) | 16.12 (-5; 37.24) |

Only chemicals that had a interaction p-value < 0.2 were investigated in the sex-stratified analysis.

Abbreviations: ∑: Molar sum; MnBP: Mono-n-butyl phthalate; MiBP: Mono-isobutyl phthalate; MBzP: Monobenzyl phthalate; DiNP: Diisononyl phthalate.

Adjustment factors: Maternal age, parity, education level, active smoking, passive smoking, BMI before pregnancy, gestational duration, maternity ward

Supplementary Table S8 –Sensitivity analysis: Adjusted associations between standardized phenols and phthalate metabolites urinary concentrations and PFR and placental weight – weighted analysis^a^

|  |  | **PFR** | | **Placental weight** | |
| --- | --- | --- | --- | --- | --- |
| **Chemical** | **Trimester** | **β (95%CI)** | **Inter. pv** | **β (95%CI)** | **Inter. pv** |
| Bisphenol A | t2 | -0.13 (-0.5; 0.24) | 0,68 | -9.53 (-23.68; 4.62) | 0,44 |
|  | t3 | -0.13 (-0.46; 0.19) | 0,29 | -6.73 (-19.31; 5.85) | 0,54 |
| Bisphenol S LOD-LOQ | t2 | -1.46 (-3.09; 0.17) | 0,37 | -58.65 (-121.9; 4.6) | 0,26 |
|  | t3 | -0.54 (-1.83; 0.76) | 0,57 | -23.67 (-73.53; 26.19) | 0,51 |
| Bisphenol S >LOQ | t2 | -0.6 (-1.37; 0.18) | 0,37 | 3.68 (-26.55; 33.91) | 0,26 |
|  | t3 | 0.27 (-0.49; 1.03) | 0,57 | 14.34 (-15.01; 43.69) | 0,51 |
| Methyl paraben | t2 | 0 (-0.22; 0.21) | 0,61 | 2.21 (-6.07; 10.49) | 0,4 |
|  | t3 | 0.04 (-0.13; 0.2) | 0,69 | 1.96 (-4.28; 8.19) | 0,55 |
| Ethyl paraben | t2 | -0.02 (-0.23; 0.18) | 0,82 | -0.3 (-8.25; 7.65) | 0,46 |
|  | t3 | 0.09 (-0.12; 0.3) | 0,19 | 3.4 (-4.71; 11.5) | 0,46 |
| Propyl paraben | t2 | 0 (-0.11; 0.1) | 0,68 | -0.56 (-4.75; 3.63) | 0,73 |
|  | t3 | 0 (-0.1; 0.1) | 0,71 | -1.23 (-5.08; 2.63) | 0,91 |
| Butyl paraben LOD-LOQ | t2 | -0.57 (-1.44; 0.3) | 0,49 | -16.08 (-49.76; 17.6) | 0,5 |
|  | t3 | -0.35 (-1.35; 0.64) | 0,6 | -27.7 (-66.02; 10.62) | 0,78 |
| Butyl paraben >LOQ | t2 | 0.5 (-0.59; 1.58) | 0,49 | -4.9 (-46.98; 37.17) | 0,5 |
|  | t3 | 0.74 (-0.27; 1.76) | 0,6 | 3.08 (-36.19; 42.35) | 0,78 |
| Benzophenone-3 | t2 | -0.01 (-0.22; 0.2) | 0,24 | -0.7 (-8.95; 7.55) | 0,17 |
|  | t3 | 0.08 (-0.17; 0.33) | 0,41 | 0.16 (-9.49; 9.8) | 0,51 |
| Triclosan | t2 | -0.09 (-0.25; 0.07) | 0,91 | -3.55 (-9.7; 2.6) | 0,63 |
|  | t3 | 0.09 (-0.07; 0.26) | 0,71 | 3.89 (-2.53; 10.32) | 0,93 |
| MEP | t2 | 0.04 (-0.27; 0.35) | 0,26 | 1.02 (-11.12; 13.16) | 0,88 |
|  | t3 | -0.01 (-0.34; 0.31) | 0,33 | -1.35 (-13.95; 11.25) | 0,33 |
| MnBP | t2 | -0.4 (-0.94; 0.13) | 0,44 | -14.58 (-35.29; 6.13) | 0,68 |
|  | t3 | -0.1 (-0.59; 0.38) | 0,01 | -0.42 (-19.05; 18.2) | 0,01 |
| MiBP | t2 | -0.08 (-0.6; 0.44) | 0,94 | -5.96 (-26.09; 14.18) | 0,92 |
|  | t3 | 0.26 (-0.2; 0.72) | 0,4 | 8.59 (-9.31; 26.5) | 0,55 |
| MBzP | t2 | -0.58 (-1.04; -0.12) | 0,82 | -22.78 (-40.5; -5.06) | 0,84 |
|  | t3 | -0.45 (-0.87; -0.03) | 0,08 | -15.87 (-31.9; 0.17) | 0,05 |
| OH-MPHP | t2 | -0.31 (-0.83; 0.21) | 0,41 | -8.54 (-28.58; 11.5) | 0,77 |
|  | t3 | 0.21 (-0.34; 0.76) | 0,3 | 7.98 (-13.35; 29.31) | 0,58 |
| ∑DEHP | t2 | -0.19 (-0.72; 0.34) | 0,92 | -10.25 (-30.58; 10.09) | 0,99 |
|  | t3 | 0.06 (-0.5; 0.62) | 0,37 | 9.25 (-12.33; 30.82) | 0,73 |
| ∑DiNP | t2 | 0.15 (-0.26; 0.56) | 0,27 | 3.83 (-12.08; 19.73) | 0,93 |
|  | t3 | -0.18 (-0.6; 0.23) | 0,01 | -2.31 (-18.31; 13.69) | 0,01 |
| ∑DINCH | t2 | 0.31 (-0.03; 0.65) | 0,11 | 11.1 (-2.06; 24.27) | 0,15 |
|  | t3 | 0.05 (-0.29; 0.4) | 0,82 | 3.05 (-10.32; 16.43) | 0,15 |

^a^ weights were define by taking the inverse of the probability of having an available placental weight measurements. This probability was computed by a logistic model for which variables were: Maternal age, parity, education level, active smoking, passive smoking, BMI before pregnancy, gestational duration, maternity ward and delivery mode

Abbreviations: ∑: Molar sum; MEP: Monoethyl phthalate; MnBP: Mono-n-butyl phthalate; MiBP: Mono-isobutyl phthalate; MBzP: Monobenzyl phthalate; OH-MPHP: 6-hydroxy-mono-propyl-heptyl phthalate; DINCH: Di(isononyl)cyclohexane-1,2-dicarboxylate; DiNP: Diisononyl phthalate; DEHP: Di(2-ethylhexyl) phthalate. Interaction p-value between the exposure and the child’s sex.

Adjustment factors: Maternal age, parity, education level, active smoking, passive smoking, BMI before pregnancy, gestational duration, maternity ward

Supplementary Table S9 -Sensitivity analysis, removal of influential values: Adjusted associations between standardized phenols and phthalates urinary concentrations and placental outcomes

|  |  | **Placental thickness T2** | | **PVR T2** | | **PVR T3** | | **PFR** | | **Placental weight** | |
| --- | --- | --- | --- | --- | --- | --- | --- | --- | --- | --- | --- |
| **Chemical** | **Trimester** | **β (95%CI)** | **Inter. pv** | **β (95%CI)** | **Inter. pv** | **β (95%CI)** | **Inter. pv** | **β (95%CI)** | **Inter. pv** | **β (95%CI)** | **Inter. pv** |
| Bisphenol A | t2 | -0.56 (-1.25; 0.13) | 0,9 | 0.38 (-0.4; 1.16) | 0,7 | -0.17 (-1.04; 0.69) | 0,71 | -0.13 (-0.5; 0.24) | 0,68 | -9.11 (-23.15; 4.94) | 0,44 |
|  | t3 | - | - | - | - | 0.28 (-0.48; 1.04) | 0,87 | -0.16 (-0.45; 0.14) | 0,29 | -6.4 (-18.12; 5.33) | 0,54 |
| Bisphenol S LOD-LOQ | t2 | -0.28 (-3.37; 2.81) | 0,15 | 0 (-3.66; 3.65) | 0,72 | 1.12 (-2.6; 4.85) | 0,64 | -1.47 (-3.09; 0.15) | 0,37 | -57.66 (-120.11; 4.79) | 0,26 |
|  | t3 | - | - | - | - | 0.45 (-2.91; 3.8) | 0,82 | -0.53 (-1.68; 0.61) | 0,57 | -24.57 (-70.78; 21.64) | 0,51 |
| Bisphenol S >LOQ | t2 | 1.18 (-0.29; 2.64) | 0,15 | 0.38 (-1.27; 2.03) | 0,72 | -0.14 (-1.97; 1.68) | 0,64 | -0.68 (-1.45; 0.1) | 0,37 | 3.21 (-26.45; 32.87) | 0,26 |
|  | t3 | - | - | - | - | -0.21 (-2.05; 1.64) | 0,82 | 0.14 (-0.57; 0.84) | 0,57 | 8.73 (-19.55; 37.01) | 0,51 |
| Methyl paraben | t2 | -0.04 (-0.46; 0.38) | 0,97 | 0.23 (-0.24; 0.7) | 0,27 | 0.3 (-0.22; 0.82) | 0,3 | 0 (-0.21; 0.22) | 0,61 | 1.73 (-6.39; 9.84) | 0,4 |
|  | t3 | - | - | - | - | -0.04 (-0.43; 0.34) | 0,27 | 0.04 (-0.11; 0.18) | 0,69 | 2.16 (-3.66; 7.98) | 0,55 |
| Ethyl paraben | t2 | 0.16 (-0.24; 0.57) | 0,98 | -0.23 (-0.67; 0.21) | 0,56 | 0.09 (-0.42; 0.59) | 0,77 | -0.04 (-0.25; 0.16) | 0,82 | -0.9 (-8.8; 7) | 0,46 |
|  | t3 | - | - | - | - | 0.39 (-0.12; 0.9) | 0,32 | 0.11 (-0.08; 0.3) | 0,19 | 4.36 (-3.3; 12.03) | 0,46 |
| Propyl paraben | t2 | -0.08 (-0.29; 0.14) | 0,86 | 0.07 (-0.18; 0.31) | 0,02 | -0.12 (-0.38; 0.15) | 0,87 | -0.01 (-0.11; 0.1) | 0,68 | -1.16 (-5.28; 2.97) | 0,73 |
|  | t3 | - | - | - | - | -0.2 (-0.43; 0.04) | 0,62 | 0.02 (-0.07; 0.11) | 0,71 | -0.79 (-4.43; 2.84) | 0,91 |
| Butyl paraben LOD-LOQ | t2 | -0.4 (-2.29; 1.5) | 0,19 | -0.92 (-2.95; 1.11) | 0,23 | -0.74 (-3; 1.51) | 0,16 | -0.5 (-1.37; 0.37) | 0,49 | -8.8 (-42.15; 24.56) | 0,5 |
|  | t3 | - | - | - | - | -0.86 (-3.3; 1.58) | 0,78 | -0.37 (-1.27; 0.53) | 0,6 | -22.46 (-58.62; 13.69) | 0,78 |
| Butyl paraben >LOQ | t2 | -0.19 (-2.11; 1.73) | 0,19 | -0.4 (-2.6; 1.81) | 0,23 | -0.87 (-3.31; 1.57) | 0,16 | 0.51 (-0.57; 1.59) | 0,49 | -2.67 (-44.29; 38.96) | 0,5 |
|  | t3 | - | - | - | - | -0.11 (-2.5; 2.28) | 0,78 | 0.86 (-0.05; 1.78) | 0,6 | 7.05 (-29.77; 43.87) | 0,78 |
| Benzophenone-3 | t2 | -0.18 (-0.58; 0.23) | 0,76 | -0.23 (-0.66; 0.21) | 0,23 | 0.22 (-0.27; 0.71) | 0,2 | -0.04 (-0.25; 0.18) | 0,24 | -1.12 (-9.26; 7.02) | 0,17 |
|  | t3 | - | - | - | - | 0.21 (-0.33; 0.75) | 0,5 | 0.07 (-0.16; 0.29) | 0,41 | 0.3 (-8.77; 9.36) | 0,51 |
| Triclosan | t2 | 0.27 (-0.04; 0.58) | 0,49 | -0.09 (-0.43; 0.25) | 0,25 | 0.12 (-0.26; 0.5) | 0,15 | -0.09 (-0.25; 0.07) | 0,91 | -3.03 (-9.11; 3.05) | 0,63 |
|  | t3 | - | - | - | - | 0.15 (-0.24; 0.54) | 0,92 | 0.05 (-0.1; 0.21) | 0,71 | 2.14 (-4.02; 8.31) | 0,93 |
| MEP | t2 | 0.39 (-0.22; 0.99) | 0,42 | -0.21 (-0.9; 0.47) | 0,43 | 0.3 (-0.45; 1.05) | 0,86 | -0.01 (-0.33; 0.3) | 0,26 | -0.75 (-12.78; 11.29) | 0,88 |
|  | t3 | - | - | - | - | 0.23 (-0.52; 0.98) | 0,19 | -0.11 (-0.41; 0.18) | 0,33 | -4.83 (-16.7; 7.04) | 0,33 |
| MnBP | t2 | 0.12 (-0.88; 1.12) | 0,22 | -1.25 (-2.34; -0.16) | 0,45 | -0.87 (-2.12; 0.37) | 0,8 | -0.39 (-0.93; 0.16) | 0,44 | -13.69 (-34.69; 7.32) | 0,68 |
|  | t3 | - | - | - | - | -1.22 (-2.41; -0.03) | 0,6 | -0.01 (-0.46; 0.43) | 0,01 | 2.63 (-15.29; 20.56) | 0,01 |
| MiBP | t2 | -0.55 (-1.53; 0.43) | 0,54 | -0.18 (-1.29; 0.94) | 0,6 | -1.28 (-2.48; -0.08) | 0,31 | -0.05 (-0.58; 0.48) | 0,94 | -4.77 (-25.02; 15.48) | 0,92 |
|  | t3 | - | - | - | - | -1.09 (-2.18; 0) | 0,06 | 0.32 (-0.11; 0.75) | 0,4 | 12 (-5.21; 29.21) | 0,55 |
| MBzP | t2 | -0.5 (-1.31; 0.31) | 0,81 | -0.49 (-1.4; 0.43) | 0,11 | -0.43 (-1.44; 0.59) | 0,46 | -0.54 (-1; -0.08) | 0,82 | -20.29 (-37.97; -2.61) | 0,84 |
|  | t3 | - | - | - | - | -0.8 (-1.77; 0.17) | 0,93 | -0.25 (-0.63; 0.13) | 0,08 | -11.5 (-26.84; 3.84) | 0,05 |
| OH-MPHP | t2 | 0.64 (-0.41; 1.69) | 0,25 | -0.46 (-1.6; 0.68) | 0,32 | -0.14 (-1.48; 1.2) | 0,44 | -0.29 (-0.83; 0.24) | 0,41 | -7.33 (-27.81; 13.15) | 0,77 |
|  | t3 | - | - | - | - | -1.05 (-2.42; 0.31) | 0,33 | 0.29 (-0.2; 0.79) | 0,3 | 9.25 (-10.47; 28.98) | 0,58 |
| ∑DEHP | t2 | -0.48 (-1.48; 0.51) | 0,73 | -0.95 (-2.08; 0.17) | 0,48 | -0.39 (-1.62; 0.84) | 0,89 | -0.17 (-0.7; 0.35) | 0,92 | -8.86 (-28.98; 11.26) | 0,99 |
|  | t3 | - | - | - | - | -0.83 (-2.18; 0.52) | 0,94 | 0.15 (-0.36; 0.66) | 0,37 | 13.61 (-6.8; 34.02) | 0,73 |
| ∑DiNP | t2 | -0.07 (-0.79; 0.66) | 0,8 | 0.19 (-0.62; 0.99) | 0,72 | -0.56 (-1.45; 0.34) | 0,03 | 0.14 (-0.27; 0.55) | 0,27 | 3.46 (-12.23; 19.15) | 0,93 |
|  | t3 | - | - | - | - | -0.32 (-1.33; 0.7) | 0,62 | -0.01 (-0.39; 0.37) | 0,01 | 3.12 (-12.21; 18.45) | 0,01 |
| ∑DINCH | t2 | -0.03 (-0.67; 0.61) | 0,86 | -0.51 (-1.23; 0.21) | 0,86 | -0.61 (-1.43; 0.21) | 0,21 | 0.33 (-0.01; 0.68) | 0,11 | 11.28 (-1.86; 24.42) | 0,15 |
|  | t3 | - | - | - | - | -0.31 (-1.12; 0.5) | 0,86 | 0.11 (-0.21; 0.42) | 0,82 | 4.24 (-8.45; 16.92) | 0,15 |

Abbreviations: ∑: Molar sum; MEP: Monoethyl phthalate; MnBP: Mono-n-butyl phthalate; MiBP: Mono-isobutyl phthalate; MBzP: Monobenzyl phthalate; OH-MPHP: 6-hydroxy-mono-propyl-heptyl phthalate; DINCH: Di(isononyl)cyclohexane-1,2-dicarboxylate; DiNP: Diisononyl phthalate; DEHP: Di(2-ethylhexyl) phthalate. Interaction p-value between the exposure and the child’s sex.

Adjustment factors: Maternal age, parity, education level, active smoking, passive smoking, BMI before pregnancy, gestational duration, maternity ward

Supplementary Table S10 – Sensitivity analysis: Adjusted associations between standardized phenols and phthalates urinary concentrations and placental outcomes, analysis additionally adjusted on specific gravity

|  |  | **Placental thickness T2** | | **PVR T2** | | **PVR T3** | | **PFR** | | **Placental weight** | |
| --- | --- | --- | --- | --- | --- | --- | --- | --- | --- | --- | --- |
| **Chemical** | **Trimester** | **β (95%CI)** | **Inter.pv** | **β (95%CI)** | **Inter.pv** | **β (95%CI)** | **Inter.pv** | **β (95%CI)** | **Inter.pv** | **β (95%CI)** | **Inter.pv** |
| Bisphenol A | t2 | -0.54 (-1.24; 0.17) | 0,88 | 0.44 (-0.35; 1.23) | 0,71 | -0.12 (-0.99; 0.76) | 0,69 | -0.07 (-0.44; 0.3) | 0,63 | -7.55 (-21.85; 6.74) | 0,41 |
|  | t3 | - | - | - | - | 0.38 (-0.41; 1.18) | 0,87 | -0.11 (-0.44; 0.22) | 0,23 | -6.99 (-19.66; 5.69) | 0,43 |
| Bisphenol S LOD-LOQ | t2 | -0.68 (-3.82; 2.46) | 0,16 | -0.1 (-3.8; 3.61) | 0,71 | 0.88 (-2.9; 4.66) | 0,65 | -1.58 (-3.19; 0.04) | 0,36 | -62.48 (-125.02; 0.06) | 0,25 |
|  | t3 | - | - | - | - | 0.13 (-3.34; 3.6) | 0,79 | -0.96 (-2.26; 0.34) | 0,42 | -37.28 (-87.44; 12.88) | 0,42 |
| Bisphenol S >LOQ | t2 | 1.2 (-0.27; 2.67) | 0,16 | 0.53 (-1.11; 2.17) | 0,71 | -0.19 (-2.02; 1.64) | 0,65 | -0.65 (-1.41; 0.12) | 0,36 | 3.12 (-26.4; 32.64) | 0,25 |
|  | t3 | - | - | - | - | -0.29 (-2.16; 1.58) | 0,79 | -0.1 (-0.87; 0.67) | 0,42 | 0.92 (-28.9; 30.74) | 0,42 |
| Methyl paraben | t2 | 0.05 (-0.37; 0.48) | 0,96 | 0.25 (-0.23; 0.72) | 0,26 | 0.35 (-0.18; 0.88) | 0,28 | 0.01 (-0.2; 0.22) | 0,57 | 2.64 (-5.57; 10.84) | 0,37 |
|  | t3 | - | - | - | - | -0.03 (-0.42; 0.37) | 0,28 | 0.06 (-0.1; 0.22) | 0,7 | 2.96 (-3.2; 9.11) | 0,6 |
| Ethyl paraben | t2 | 0.28 (-0.13; 0.69) | 1 | -0.19 (-0.64; 0.26) | 0,56 | 0.12 (-0.39; 0.63) | 0,79 | -0.01 (-0.22; 0.19) | 0,84 | 0.14 (-7.83; 8.12) | 0,45 |
|  | t3 | - | - | - | - | 0.46 (-0.08; 1) | 0,3 | 0.12 (-0.1; 0.33) | 0,22 | 5 (-3.25; 13.25) | 0,54 |
| Propyl paraben | t2 | -0.06 (-0.28; 0.15) | 0,81 | 0.07 (-0.18; 0.31) | 0,02 | -0.11 (-0.38; 0.15) | 0,89 | -0.01 (-0.12; 0.1) | 0,61 | -1.01 (-5.14; 3.11) | 0,67 |
|  | t3 | - | - | - | - | -0.2 (-0.44; 0.04) | 0,61 | -0.01 (-0.11; 0.09) | 0,73 | -1.26 (-5.08; 2.57) | 0,89 |
| Butyl paraben LOD-LOQ | t2 | -0.01 (-1.87; 1.86) | 0,19 | -0.88 (-2.92; 1.16) | 0,24 | -0.71 (-2.97; 1.54) | 0,15 | -0.42 (-1.29; 0.44) | 0,49 | -7.4 (-40.75; 25.95) | 0,5 |
|  | t3 | - | - | - | - | -0.87 (-3.35; 1.62) | 0,81 | -0.21 (-1.2; 0.77) | 0,37 | -22.31 (-60.32; 15.7) | 0,59 |
| Butyl paraben >LOQ | t2 | -0.22 (-2.14; 1.71) | 0,19 | -0.4 (-2.61; 1.81) | 0,24 | -0.88 (-3.32; 1.56) | 0,15 | 0.55 (-0.52; 1.62) | 0,49 | -2.48 (-44; 39.04) | 0,5 |
|  | t3 | - | - | - | - | -0.16 (-2.62; 2.29) | 0,81 | 0.59 (-0.42; 1.6) | 0,37 | -0.86 (-39.86; 38.15) | 0,59 |
| Benzophenone-3 | t2 | -0.16 (-0.56; 0.25) | 0,81 | -0.21 (-0.65; 0.23) | 0,22 | 0.26 (-0.24; 0.76) | 0,21 | 0.01 (-0.2; 0.23) | 0,23 | -0.17 (-8.47; 8.13) | 0,17 |
|  | t3 | - | - | - | - | 0.24 (-0.31; 0.8) | 0,47 | 0.1 (-0.15; 0.35) | 0,42 | 0.91 (-8.76; 10.57) | 0,49 |
| Triclosan | t2 | 0.29 (-0.02; 0.6) | 0,52 | -0.08 (-0.42; 0.25) | 0,25 | 0.13 (-0.25; 0.51) | 0,16 | -0.07 (-0.23; 0.09) | 0,98 | -2.71 (-8.79; 3.36) | 0,69 |
|  | t3 | - | - | - | - | 0.16 (-0.24; 0.56) | 0,99 | 0.11 (-0.05; 0.28) | 0,53 | 4.98 (-1.43; 11.39) | 0,8 |
| MEP | t2 | 0.55 (-0.08; 1.17) | 0,34 | -0.17 (-0.88; 0.54) | 0,46 | 0.41 (-0.37; 1.18) | 0,94 | 0.04 (-0.28; 0.36) | 0,22 | 0.88 (-11.52; 13.27) | 0,82 |
|  | t3 | - | - | - | - | 0.41 (-0.42; 1.24) | 0,2 | 0.04 (-0.31; 0.39) | 0,33 | -1.21 (-14.61; 12.19) | 0,34 |
| MnBP | t2 | 0.44 (-0.66; 1.55) | 0,27 | -1.41 (-2.61; -0.2) | 0,48 | -0.79 (-2.17; 0.58) | 0,79 | -0.26 (-0.85; 0.33) | 0,46 | -10.04 (-32.71; 12.62) | 0,7 |
|  | t3 | - | - | - | - | -1.42 (-2.84; 0.01) | 0,63 | 0.08 (-0.51; 0.67) | 0 | 4.5 (-18.17; 27.17) | 0,01 |
| MiBP | t2 | -0.37 (-1.44; 0.69) | 0,49 | -0.19 (-1.39; 1.01) | 0,58 | -1.26 (-2.56; 0.03) | 0,31 | 0.14 (-0.43; 0.71) | 0,9 | 0.54 (-21.45; 22.53) | 0,9 |
|  | t3 | - | - | - | - | -1.39 (-2.69; -0.09) | 0,06 | 0.61 (0.06; 1.15) | 0,38 | 17.23 (-3.91; 38.36) | 0,48 |
| MBzP | t2 | -0.45 (-1.29; 0.39) | 0,87 | -0.45 (-1.39; 0.5) | 0,1 | -0.34 (-1.39; 0.72) | 0,49 | -0.48 (-0.95; 0) | 0,85 | -18.56 (-36.74; -0.38) | 0,86 |
|  | t3 | - | - | - | - | -0.86 (-1.93; 0.22) | 0,91 | -0.42 (-0.87; 0.04) | 0,07 | -17.19 (-34.56; 0.18) | 0,04 |
| OH-MPHP | t2 | 0.82 (-0.26; 1.91) | 0,25 | -0.41 (-1.58; 0.76) | 0,32 | 0 (-1.38; 1.38) | 0,46 | -0.23 (-0.77; 0.31) | 0,44 | -5.25 (-26.01; 15.51) | 0,81 |
|  | t3 | - | - | - | - | -1.02 (-2.47; 0.43) | 0,33 | 0.3 (-0.26; 0.87) | 0,19 | 10.48 (-11.32; 32.29) | 0,46 |
| ∑DEHP | t2 | -0.35 (-1.41; 0.7) | 0,65 | -0.93 (-2.11; 0.25) | 0,48 | -0.25 (-1.55; 1.06) | 0,93 | -0.06 (-0.61; 0.48) | 0,87 | -5.53 (-26.63; 15.58) | 0,95 |
|  | t3 | - | - | - | - | -0.75 (-2.28; 0.78) | 0,98 | 0.25 (-0.36; 0.87) | 0,39 | 15.78 (-7.97; 39.53) | 0,72 |
| ∑DiNP | t2 | 0.12 (-0.63; 0.88) | 0,83 | 0.27 (-0.56; 1.1) | 0,7 | -0.49 (-1.42; 0.43) | 0,04 | 0.21 (-0.2; 0.63) | 0,3 | 5.79 (-10.18; 21.77) | 0,98 |
|  | t3 | - | - | - | - | -0.22 (-1.32; 0.88) | 0,61 | -0.21 (-0.65; 0.23) | 0 | -1.92 (-18.79; 14.95) | 0,01 |
| ∑DINCH | t2 | 0.07 (-0.59; 0.73) | 0,86 | -0.48 (-1.23; 0.26) | 0,86 | -0.56 (-1.4; 0.28) | 0,21 | 0.42 (0.07; 0.77) | 0,1 | 14.91 (1.32; 28.5) | 0,13 |
|  | t3 | - | - | - | - | -0.22 (-1.09; 0.64) | 0,87 | 0.14 (-0.23; 0.5) | 0,93 | 5.61 (-8.34; 19.55) | 0,2 |

Abbreviations: ∑: Molar sum; MEP: Monoethyl phthalate; MnBP: Mono-n-butyl phthalate; MiBP: Mono-isobutyl phthalate; MBzP: Monobenzyl phthalate; OH-MPHP: 6-hydroxy-mono-propyl-heptyl phthalate; DINCH: Di(isononyl)cyclohexane-1,2-dicarboxylate; DiNP: Diisononyl phthalate; DEHP: Di(2-ethylhexyl) phthalate. Interaction p-value between the exposure and the child’s sex.

Adjustment factors: Maternal age, parity, education level, active smoking, passive smoking, BMI before pregnancy, gestational duration, maternity ward

Supplementary Table S11 - Adjusted associations between standardized phenols and phthalates urinary concentrations and placental outcomes, not adjusted on gestational age.

|  |  | **PFR** | | **Placental weight** | |
| --- | --- | --- | --- | --- | --- |
| Chemical | Trimester | β (95%CI) | Inter. pv | β (95%CI) | Inter. pv |
| Bisphenol A | t2 | -0.09 (-0.46; 0.27) | 0,64 | -10.21 (-24.34; 3.93) | 0,49 |
|  | t3 | -0.16 (-0.48; 0.16) | 0,33 | -7.95 (-20.31; 4.41) | 0,49 |
| Bisphenol S LOD-LOQ | t2 | -1.49 (-3.11; 0.13) | 0,36 | -54.34 (-117.35; 8.67) | 0,28 |
|  | t3 | -0.7 (-1.97; 0.57) | 0,63 | -23.51 (-72.28; 25.25) | 0,45 |
| Bisphenol S >LOQ | t2 | -0.68 (-1.45; 0.09) | 0,36 | 5.92 (-23.97; 35.81) | 0,28 |
|  | t3 | 0.05 (-0.73; 0.83) | 0,63 | 6.91 (-22.95; 36.76) | 0,45 |
| Methyl paraben | t2 | -0.02 (-0.23; 0.2) | 0,52 | 1.78 (-6.42; 9.97) | 0,53 |
|  | t3 | 0.04 (-0.12; 0.2) | 0,69 | 1.87 (-4.27; 8.02) | 0,56 |
| Ethyl paraben | t2 | -0.05 (-0.26; 0.15) | 0,92 | 0.13 (-7.83; 8.09) | 0,57 |
|  | t3 | 0.07 (-0.14; 0.28) | 0,29 | 3.85 (-4.25; 11.94) | 0,33 |
| Propyl paraben | t2 | -0.02 (-0.12; 0.09) | 0,74 | -0.85 (-5.02; 3.32) | 0,65 |
|  | t3 | -0.01 (-0.11; 0.09) | 0,75 | -1.16 (-5; 2.67) | 0,86 |
| Butyl paraben LOD-LOQ | t2 | -0.35 (-1.22; 0.51) | 0,38 | -13.84 (-47.35; 19.66) | 0,66 |
|  | t3 | -0.25 (-1.25; 0.74) | 0,59 | -26.66 (-64.78; 11.46) | 0,8 |
| Butyl paraben >LOQ | t2 | 0.5 (-0.58; 1.58) | 0,38 | -0.29 (-42.28; 41.7) | 0,66 |
|  | t3 | 0.61 (-0.4; 1.61) | 0,59 | 8.5 (-30.18; 47.17) | 0,8 |
| Benzophenone-3 | t2 | -0.01 (-0.23; 0.2) | 0,26 | -1.49 (-9.72; 6.74) | 0,16 |
|  | t3 | 0.08 (-0.17; 0.32) | 0,44 | -0.16 (-9.73; 9.41) | 0,49 |
| Triclosan | t2 | -0.08 (-0.24; 0.08) | 0,9 | -2.91 (-9.04; 3.22) | 0,66 |
|  | t3 | 0.09 (-0.08; 0.26) | 0,65 | 5.09 (-1.32; 11.51) | 1 |
| MEP | t2 | 0 (-0.31; 0.31) | 0,34 | -2.06 (-14.18; 10.06) | 0,72 |
|  | t3 | -0.09 (-0.41; 0.24) | 0,29 | -1.75 (-14.23; 10.73) | 0,39 |
| MnBP | t2 | -0.34 (-0.89; 0.21) | 0,53 | -15.34 (-36.49; 5.8) | 0,56 |
|  | t3 | -0.14 (-0.63; 0.34) | 0,01 | -0.81 (-19.65; 18.04) | 0,02 |
| MiBP | t2 | 0.04 (-0.49; 0.56) | 0,88 | -8.3 (-28.52; 11.92) | 1 |
|  | t3 | 0.28 (-0.19; 0.75) | 0,39 | 7.07 (-11.04; 25.19) | 0,58 |
| MBzP | t2 | -0.51 (-0.97; -0.05) | 0,8 | -20.95 (-38.78; -3.13) | 0,86 |
|  | t3 | -0.51 (-0.92; -0.1) | 0,07 | -15.19 (-31.1; 0.72) | 0,06 |
| OH-MPHP | t2 | -0.31 (-0.84; 0.23) | 0,43 | -6.6 (-27.29; 14.1) | 0,74 |
|  | t3 | 0.09 (-0.45; 0.62) | 0,37 | 9.67 (-10.98; 30.33) | 0,5 |
| ∑DEHP | t2 | -0.19 (-0.71; 0.34) | 0,86 | -8.39 (-28.72; 11.94) | 0,94 |
|  | t3 | -0.02 (-0.58; 0.53) | 0,37 | 11.89 (-9.47; 33.25) | 0,75 |
| ∑DiNP | t2 | 0.12 (-0.29; 0.53) | 0,23 | 4.65 (-11.19; 20.5) | 0,97 |
|  | t3 | -0.27 (-0.69; 0.14) | 0,01 | -0.66 (-16.64; 15.32) | 0,01 |
| ∑DINCH | t2 | 0.33 (-0.01; 0.67) | 0,11 | 11.12 (-2.16; 24.4) | 0,15 |
|  | t3 | 0.02 (-0.33; 0.37) | 0,7 | 2.72 (-10.66; 16.1) | 0,21 |

Abbreviations: ∑: Molar sum; MEP: Monoethyl phthalate; MnBP: Mono-n-butyl phthalate; MiBP: Mono-isobutyl phthalate; MBzP: Monobenzyl phthalate; OH-MPHP: 6-hydroxy-mono-propyl-heptyl phthalate; DINCH: Di(isononyl)cyclohexane-1,2-dicarboxylate; DiNP: Diisononyl phthalate; DEHP: Di(2-ethylhexyl) phthalate. Interaction p-value between the exposure and the child’s sex.

Adjustment factors: Maternal age, parity, education level, active smoking, passive smoking, BMI before pregnancy, gestational duration, maternity ward

Supplementary Table S12 – BKMR Models’ Post-Integration Probabilities (PIPs)

| **Chemical** | **Placental thickness T2** | **PVR T2** | **PVR T3** | **PFR** | **Placental weight** |
| --- | --- | --- | --- | --- | --- |
| **2^nd^ Trimester** |  |  |  |  |  |
| Bisphenol A | 0,17 | 0,01 | 0,96 | 0,01 | 0,05 |
| Methyl paraben | 0,01 | 0 | 0 | 0,01 | 0,02 |
| Ethyl paraben | 0,03 | 0,01 | 0 | 0 | 0,01 |
| Propyl paraben | 0 | 0 | 0 | 0 | 0 |
| Triclosan | 0,11 | 0 | 0 | 0 | 0,01 |
| Benzophenone-3 | 0,02 | 0 | 0 | 0,02 | 0,02 |
| MEP | 0,06 | 0,18 | 0 | 0,01 | 0,02 |
| OH-MPHP | 0,1 | 0,03 | 0 | 0,07 | 0,05 |
| MBzP | 0,12 | 0,05 | 0 | 0,5 | 0,39 |
| MiBP | 0,12 | 0,03 | 0,02 | 0,06 | 0,05 |
| MnBP | 0,05 | 0,5 | 0,01 | 0,09 | 0,12 |
| DEHP | 0,1 | 0,15 | 0 | 0,04 | 0,06 |
| DiNP | 0,04 | 0,02 | 0 | 0,03 | 0,04 |
| DINCH | 0,06 | 0,02 | 0 | 0,15 | 0,15 |
| **3^rd^ Trimester** |  |  |  |  |  |
| Bisphenol A | - | - | 0,01 | 0,01 | 0,06 |
| Methyl paraben | - | - | 0 | 0 | 0,01 |
| Ethyl paraben | - | - | 0,02 | 0,01 | 0,03 |
| Propyl paraben | - | - | 0 | 0 | 0,01 |
| Triclosan | - | - | 0 | 0 | 0,01 |
| Benzophenone-3 | - | - | 0,01 | 0,03 | 0,03 |
| MEP | - | - | 0 | 0,08 | 0,06 |
| OH-MPHP | - | - | 0,61 | 0,04 | 0,06 |
| MBzP | - | - | 0,03 | 0,44 | 0,25 |
| MiBP | - | - | 0,15 | 0,06 | 0,08 |
| MnBP | - | - | 0,1 | 0,07 | 0,06 |
| DEHP | - | - | 0,05 | 0,09 | 0,24 |
| DiNP | - | - | 0,01 | 0,14 | 0,07 |
| DINCH | - | - | 0,01 | 0,03 | 0,03 |

Second trimester outcomes (placental thickness and Placental vascular resistance (PVR) T2) had a mixture comprised only of second trimester exposures. The others outcomes had a mixture composed of both trimester second and third trimester exposures. Abbreviation: MEP: Methylethyl phthalate; OH-MPHP: 6-Hydroxy Monopropylheptylphthalate; MBzP: Monobenzyle phthalate; MiBP: Mono isobutyl phthalate; MnBP: Mono-n-butyl phthalate; DEHP: Di (2-ethylhexyl) phthalate; DiNP: Di-isononyl phthalate; DINCH: Di(isononyl)cyclohexane-1,2-dicarboxylate;
